# Lymph Flow Induces the Postnatal Formation of Mature and Functional Meningeal Lymphatic Vessels

**DOI:** 10.3389/fimmu.2019.03043

**Published:** 2020-01-14

**Authors:** László Bálint, Zsombor Ocskay, Bálint András Deák, Petra Aradi, Zoltán Jakus

**Affiliations:** ^1^Department of Physiology, Semmelweis University School of Medicine, Budapest, Hungary; ^2^MTA-SE “Lendület” Lymphatic Physiology Research Group, Hungarian Academy of Sciences and Semmelweis University, Budapest, Hungary

**Keywords:** lymphatics, meninges, developmental program, lymphatic function, lymph flow, central nervous system, macromolecule transport

## Abstract

Recently, the presence of lymphatics has been demonstrated and characterized in the dura mater, which is in contrast to the well-accepted view indicating the lack of a classical lymphatic drainage system of the central nervous system (CNS). Moreover, the role of meningeal lymphatics in the pathogenesis of Alzheimer's disease and multiple sclerosis was suggested. However, the possible regulators of the developmental program and function of meningeal lymphatics remain unclear. Here, we aimed at characterizing the lymph flow dependence of the developmental program and function of the meningeal lymphatics. First, we demonstrated that lymphatics present in the dura mater are involved in the uptake and transport of macromolecules from the CNS. Meningeal lymphatics develop during the postnatal period which process involves the maturation of the vessels. The formation of mature meningeal lymphatics coincides with the increase of the drainage of macromolecules from the CNS to the deep cervical lymph nodes. Importantly, the structural remodeling and maturation of meningeal lymphatics is impaired in *Plc*γ*2*^−/−^ mice with reduced lymph flow. Furthermore, macromolecule uptake and transport by the meningeal lymphatics are also affected in *Plc*γ*2*^−/−^ mice. Collectively, lymph flow-induced mechanical forces are required for the postnatal formation of mature and functional meningeal lymphatic vessels. Defining lymph flow-dependence of the development and function of meningeal lymphatics may lead to better understanding of the pathogenesis of neurological diseases including Alzheimer's disease and multiple sclerosis.

## Introduction

Until recently, the classical, well-accepted view was that the central nervous system (CNS) lacks lymphatic structures (1). However, sporadic studies suggested the presence of lymphatic vessels in the dura mater. The first known report about the presence of meningeal lymphatic structures was published in 1787 by Mascagni, which was followed by others during the previous decades (2–5). However, these early studies were not able to change the classical view. In 2015 two parallel studies described the presence of lymphatic structures in the dura mater in mouse models (6, 7). In addition, the meningeal lymphatics were shown in non-human primates and humans, in which the morphological characteristics of these lymphatic vessels were very similar to the structure of meningeal lymphatics in rodents (8, 9). Importantly, the latest studies using mouse models showed the possible role of meningeal lymphatics in the pathogenesis of neurological diseases affecting the CNS including Alzheimer's disease and neuroinflammatory diseases such as multiple sclerosis (10–14). These studies suggested the importance of meningeal lymphatics not only under physiological conditions but also in the pathophysiology of the CNS.

It is known that macromolecules are drained to the deep cervical lymph nodes from the CNS (6, 7, 15–17). The classical concept of the possible drainage routes to the cervical region is that macromolecules and immune cells leave the CNS via the cribriform plate and in the wall of the incoming and outgoing blood vessels and nerves of the brain (18). Recent studies suggested that meningeal lymphatic vessels are involved (or might be involved) in the transport of macromolecules and immune cells from the CNS (6, 7, 10, 11, 19). In parallel, another report demonstrated that macromolecules injected into the CNS are drained to the deep cervical lymph nodes via several different transport routes including the paravascular space of the pia mater and perineural routes (e.g., next to the optic nerve), but the involvement of meningeal lymphatics in the uptake and transport of macromolecules from the CNS was unclear based on their work (20).

It has recently been revealed that meningeal lymphatics develop during the postnatal period, and the Vascular endothelial growth factor C (VEGFC)—Vascular endothelial growth factor receptor 3 (VEGFR3) signaling axis, similarly to the developmental program in other organs including the gastrointestinal tract, is required for the development and structural maintenance of these vessels (21–23). However, the possible other regulators of the developmental program and function of meningeal lymphatics remain unclear.

Maintaining lymph flow is critical for mediating the functions of the lymphatic system including fluid transport, uptake of macromolecules from the interstitial compartment and immune cell trafficking (24, 25). It has been established that mechanical forces generated by fluid shear stress regulate gene expression and lymph vessel formation *in vitro* by their direct effect on lymphatic endothelial cells (LECs) (26). The *in vivo* importance of mechanical forces in lymphatic growth and function is also suggested by human data because patients who carry mutations in the PIEZO1 mechanosensor protein expressed on the surface of LECs develop primary lymphedema (27, 28). Moreover, genetic studies in mouse models revealed the importance of PIEZO1-induced mechanical forces in the development and maintenance of lymphatics (29, 30). These reports indicate the importance and function of mechanical forces in lymphatic development, but defining the role of lymph flow-induced mechanical forces has great limitations in *in vivo* experiments.

In mouse models lacking the components of the CLEC2, SYK, SLP76 signaling axis in platelets backflow of blood is present from the venous system into the lymphatic vasculature, which phenotype develops because of the loss of platelet activation by LECs at the lympho-venous junction, where the thoracic duct meets the subclavian vein (31–33). It is known that PLCγ2 is a member of the same signaling pathway, in addition PLCγ2-deficient mice exhibit similar phenotype (blood-filled lymphatics in embryos), which is present in CLEC2-deficient, SYK-deficient and SLP76-deficient animals (31–35). Prior studies used CLEC2-deficient mice to demonstrate the role of lymphatic function and lymph flow for inducing the structural remodeling of the mesenteric lymphatics during the development of the system, which process involves the maturation of these lymphatic structures (25, 36). Moreover, in a recent report CLEC2-deficient mouse strain with reduced lymphatic function was also applied as an *in vivo* model to define the role of pulmonary lymphatics in the postnatal lung (37). It is thought that as CLEC2-deficient mice which were used in previous reports to characterize the importance of lymph flow and lymphatic function in the gastrointestinal tract and lungs, the PLCγ2-deficient system can be a perfect model to define the possible role of lymph flow in the other organs *in vivo* (25, 36, 37). It is yet to be established whether lymph flow-generated mechanical forces are involved in the morphogenesis of the lymphatic vasculature in other organs including the lymphatic vessels of the dura mater.

Here, we aimed at characterizing the lymph flow dependence of the developmental program and function of the meningeal lymphatics. Using genetic models we demonstrated that meningeal lymphatics present in the dura mater are involved in the uptake and transport of macromolecules injected into the CNS. Lymph flow mediated maturation of meningeal lymphatics occurs during the postnatal period, which process coincides with the increase of the drainage of macromolecules from the CNS. Importantly, our studies using PLCγ2-deficient mice revealed that lymph flow-induced mechanical forces are required for the postnatal formation of mature and functional meningeal lymphatic vessels.

## Materials and Methods

### Animals

Male and female C57BL/6 wild type (purchased from commercial sources), *Prox1*^*GFP*^ obtained from the Mutant Mouse Regional Resource Centers (MMRRC) and *Flt4*^*YFP*^ generously provided by Jean-Léon Thomas (INSERM, France) lymphatic endothelial cell reporter mice were used (38, 39). *Prox1*^*GFP*^ mice were maintained in heterozygous form and genotyped by a transgene-specific PCR using 5′-GAT GTG CCA TAA ATC CCA GAG CCT AT−3′ forward and 5′-GGT CGG GGT AGC GGC TGA A−3′ reverse primers, *Flt4*^*YFP*^ mice were bred in heterozygous form, and genotyped by transgene-specific PCR primer sets including 5′-GGA TCA CTC TCG GCA TGG AC−3′ forward and 5′-GGG CGT CCT CAT ACC TAG GT−3′ reverse primers.

To study the possible role of lymphatic function and lymph flow we used *Plc*γ*2*^−/−^ [generously provided by James Ihle (St. Jude Children's Research Hospital) (34)] and littermate control mice. The strain was maintained in heterozygous form and genotyped by allele-specific PCR reaction using 5′-GCC TCT GCA CAG CAC ACA TAT GG−3′ WT-specific and 5′-CAA GGT GAG ATG ACA GGA GAT CC−3′ mutant-specific forward primers along with the 5′-TTC ACC GCA TCC TCC TTT GAG TCC−3′ common reverse primer.

Experimental animals were housed in either specific pathogen free or conventional animal facilities. All animal experiments were approved by the Animal Experimentation Review Board of the Semmelweis University and the Government Office for Pest County (Hungary).

### Tissue Isolation and Fixation

To study the developmental program of the meningeal lymphatics, mice at different ages [from postnatal day 0 (P0) to P21] were used. The procedure for meninges isolation was based on the protocol of Louveau and Kipnis as described before (6, 40). Briefly, adult mice and pups older than P8 were perfused with 10 ml of Phosphate buffered saline (PBS) followed by 10 ml of 4% paraformaldehyde (PFA). Pups between P0 and P8 were perfused with 5 ml of PBS followed by 5 ml of 4% PFA. Using angled scissors, skin and muscles were removed from the skull followed by the dissection of optic nerves, eyeballs, mandibles, and muscles connecting to the lower jaw. After that the lower orbits and nasal bone were cut and the lower portion of the skull was removed. Meninges attached to the skull cap were then fixed in 4% PFA overnight at 4°C and washed in PBS.

For histological analysis dissected lymph nodes were fixed overnight in 4% PFA and washed in PBS followed by a paraffin-based histology protocol.

### Histology Procedures and Immunostaining of Histology Slides

For histological analysis, fixed lymph nodes were dehydrated in 100% ethanol and embedded in paraffin (Leica) using a Leica EG1150 tissue embedder. 7-μm-thick sections using a HM340E Thermo Scientific microtome were processed for hematoxylin-eosin (HE) (Leica) and immunohistochemistry staining as described (41). For immunohistochemistry of histology slides the following antibodies were used: anti-TER-119 (R&D Systems, MAB1125) and Alexa Fluor 488 conjugated goat anti-rat IgG antibody (Life Technologies A11006). Stained slides were mounted with Vectashield DAPI Mounting Medium (Vector Laboratories, H-1200). Tissue section samples were imaged using a Nikon Ni-U upright microscope (Nikon Instruments) using a 40x dry objective connected to a Nikon DS-Ri2 camera.

### Whole-Mount Immunostaining of Meninges, Image Acquisition and Analysis

Dissected meninges were incubated in PBS containing 10% of goat or horse serum for 1 h at room temperature followed by staining with antibodies against LYVE-1 (R&D Systems, AF2125), PROX-1 (Abcam, ab76696), PDPN (Abcam, ab92319), PECAM (R&D Systems, MAB3628), F4/80 (BioLegend, 123101). For the visualization of GFP and YFP in our transgenic lymphatic reporter mouse models anti-GFP antibody (Life Technologies, A11122) was used. All primary antibodies were diluted at 1:170 in the presence of 10% serum and 0.1% Tween 20, and were incubated for 24 h at 4°C. Thereafter, samples were incubated with Alexa Fluor 488/568/594 anti-rabbit/anti-goat/anti-rat/anti-syrian hamster IgG antibodies (Life Technologies, A21206, A11055, A11057, A11006, A11077, A21113) at 1:1000. Then whole-mount tissues were washed in PBS. Control immunostainings were used for all primary antibodies as it is represented in Supplementary Figure 1.

Images of whole-mount samples were acquired by a Nikon SMZ-25 stereo microscope (Nikon Instruments) connected to a Nikon DS-Ri2 camera or a Nikon Eclipse Ti2 microscope (Nikon Instruments) using a 10x dry objective connected to a Yokogawa CSU-W1 confocal scanner unit (Yokogawa Electric Corporation). Quantitative assessments including the length and area measurements were performed using NIS Elements software.

Structure of the meningeal lymphatic vessels was assessed using a clinical score system on a 0–15 scale by two independent investigators blinded for all parameters (genotype, age etc.) of the mice. The scoring system is based on continuity of meningeal lymphatic vessels (0–4 points; 0 point: totally discontinuous lymphatic structure; 4 points: continuous lymphatic structures); structural malformations of the meningeal lymphatics (0–3 points; 0 point: numerous severe structural malformations; 3 points: intact meningeal lymphatic structures); presence of lymphatic vessels adjacent to the superior sagittal sinus (0–2 points; 0 point: no lymphatic vessels adjacent to the superior sagittal sinus; 1 point: lymphatic vessels on one side of the superior sagittal sinus; 2 points: lymphatic vessels on both sides of the superior sagittal sinus), transverse sinus (0–2 points; 0 point: no lymphatic vessels adjacent to the transverse sinus; 1 point: lymphatic vessels adjacent to the transverse sinus on one side of the skull; 2 points: lymphatic vessels adjacent to the transverse sinus on both sides of the skull), and the middle meningeal arteries (0–2 points; 0 point: no lymphatic vessels adjacent to the middle meningeal arteries; 1 point: lymphatic vessels adjacent to the middle meningeal arteries on one side of the skull; 2 points: lymphatic vessels adjacent to the middle meningeal arteries on both sides of the skull); and number of branching points (0–2 points; 0: no branches or max. 3 branches; 1 point: 3 to 10 branches; 2 points: more than 10 branches). Mean of the values given by the two independent investigators was used for each mouse.

### Characterization of Meningeal Lymphatic Function

For monitoring meningeal lymphatic function 2.5 μl of 70 kDa Rhodamine-labeled dextran (RhD) (Life Technologies, D1818), 40 kDa RhD (Life Technologies, D1842), 3 kDa RhD (Life Technologies, D3308) from a 10 mg/ml stock and PBS were injected into the dorsal prefrontal cortex of the right hemisphere, cisterna magna or subcutaneously to mice anesthetized with 2.5% Avertin (Sigma-Aldrich, T48402). For intraparenchymal injection, a midline skin incision was made to reveal the skull bone, which was thinned 2 mm lateral and 1.5 mm caudal to the bregma. 2.5 μl of the tracers were slowly injected into ~2 mm depth with a Hamilton syringe using a blunt-ended 30G needle for 3 min. The needle was slowly removed after the mouse was left in position for 5 min to prevent leakage. For intra cisterna magna injection, the neck muscles were bluntly dissected through a small midline incision and 70 kDa RhD was slowly injected into the cisterna magna with a Hamilton syringe using a sharp-ended 32G needle for 3 min. The needle was slowly removed after the mouse was left in position for 5 min to prevent leakage. Subcutaneous injection was performed at the same injection site which was used for the intraparenchymal injection. The injection of the macromolecules was rigorously controlled in each experiment. Importantly, the injection site was carefully examined in every experimental animal. If the injection was not successful, the mouse was excluded from the dataset. For intraparenchymal injection of E17.5 embryos, pregnant female mice were anesthetized and 2.5 μl of 70 kDa RhD was injected through the uterine wall into the dorsal prefrontal cortex of the right hemisphere of each embryo by a 30G needle. Needle was removed 2 min post injection. Experimental mice or embryos between E17.5 and P21 were sacrificed 100 min post injection and drainage of the injected labeled macromolecules to the meningeal lymphatic vessels and lymph nodes in the cervical region was assessed by fluorescent stereo microscopy in combination with LEC-specific immunostaining. Drainage of the fluorescently labeled molecules into the cervical lymph nodes were quantified by measuring the mean fluorescent intensity of the area of the lymph nodes (mean fluorescent intensity of background subtracted from the mean fluorescent intensity of lymph nodes) using NIS Elements software. The drainage to the cervical area was also assessed in live mice.

### Characterization of the Gut Phenotype of *Plcγ*2^−/−^ and Littermate Control Mice

To characterize the gut lymphatic phenotype of PLCγ2-deficient and littermate control mice a clinical score system was used on a 0–4 scale by two independent investigators blinded for all parameters (genotype, age etc.) of the mice. The following parameters were included: the blood filling and structural malformations of lymphatic vessels in the mesentery and small intestine (0 or 1 point for each parameter). For quantification a mean of the values given by the two independent investigators was used for each mouse. *Plc*γ*2*^−/−^ mice with points more than 2.5 were considered as displaying severe gut phenotype.

### Monitoring the Presence of Blood in the Regional Lymph Nodes of *Plcγ*2^−/−^ and Littermate Control Mice

To monitor the presence of blood in the regional lymph nodes a score system was used on a 0–4 scale by two independent investigators blinded for all parameters (genotype, age etc.) of the mice (0–no blood in the lymph node, 4–total area of lymph node is filled with blood). Mean of the values given by the two independent investigators was used for each mouse.

### Characterization of Lymphatic Function in the Small Intestine of *Plcγ*2^−/−^ and Littermate Control Mice

To monitor lymphatic function in the small intestine, mice were fasted overnight followed by feeding them with 100 μl of 4,4-Difluoro-5,7-Dimethyl-4-Bora-3a,4a-Diaza-s-Indacene-3-Hexadecanoic Acid (BODIPY C_16_) (Thermo Fisher, D3821) diluted in sunflower oil (Sigma-Aldrich, S5007) at 1:250. Two hours later, mice were sacrificed and imaged by fluorescent stereo microscopy.

### Monitoring the Lymphatic Function in the Hind Limb of *Plcγ*2^−/−^ and Littermate Control Mice

To monitor lymphatic function in the hind limb, subcutaneous injection of 2.5 μl 10 mg/ml 70 kDa RhD was performed into the hind limb paws of young adult mice anesthetized with 2.5% Avertin (41). Mice were sacrificed 100 min post injection and drainage of the injected labeled macromolecules was assessed by fluorescent stereo microscopy.

### Presentation of the Data and Statistical Analysis

Experiments were performed the indicated number of times. For all experiments, investigators were blinded for the origin of mice and treatment from the time of euthanasia to the end of the analysis. Quantitative graphs show mean and SEM. Statistical analyses were performed using Graph Pad Prism 7.0 and Microsoft Office Excel software programs. Specific statistical tests are presented in the figure legend for each experiment. *P-*values below 0.05 were considered statistically significant.

## Results

### Lymphatic Vessels Are Present in the Dura Mater

**Figure 1:**
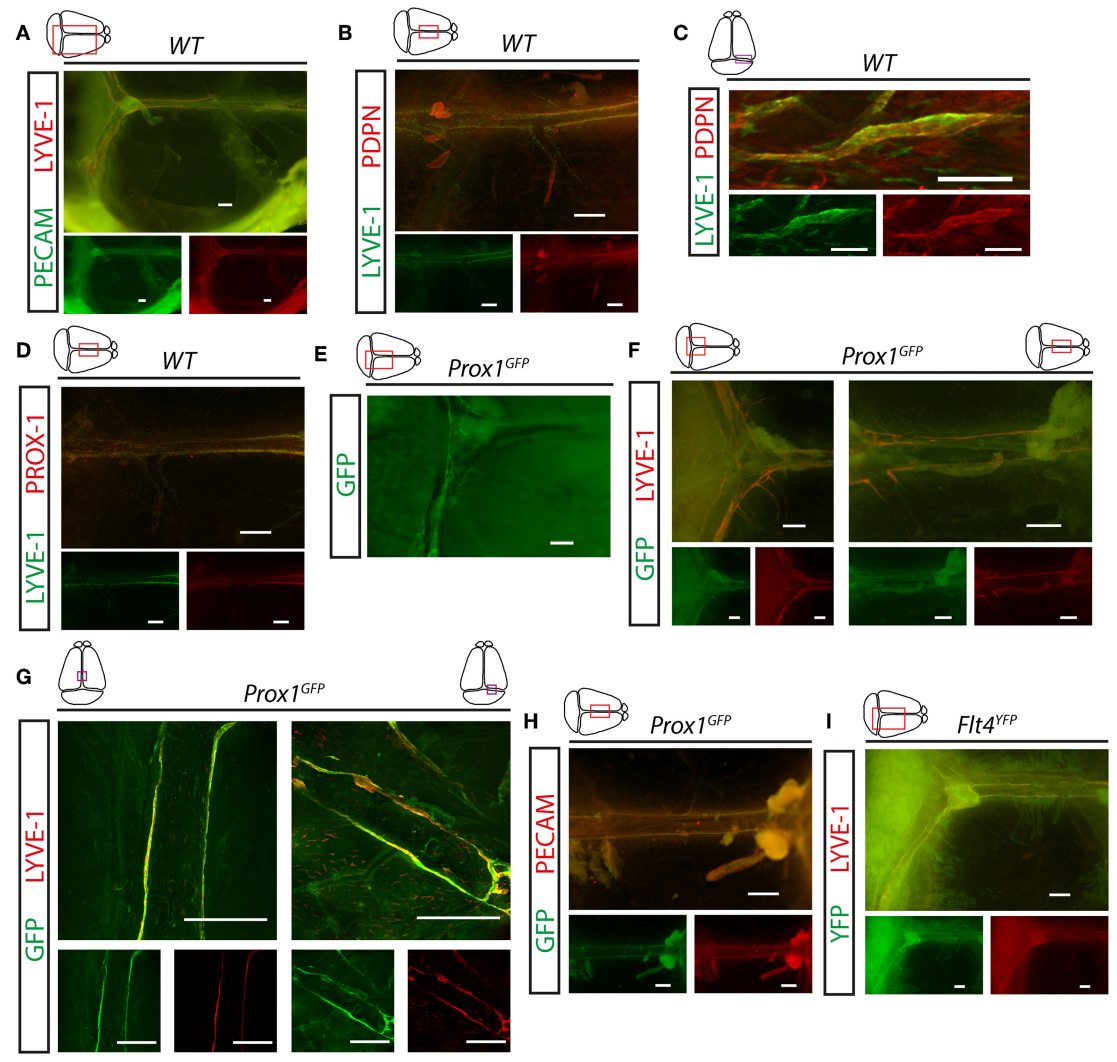
Expression of lymphatic markers in meninges of young adult mice. **(A)** Expression pattern of PECAM and LYVE-1 shown in the dura mater of young adult wild type mice (*n* = 7). Bars, 500 μm. **(B,C)** LYVE-1 and PDPN immunostaining of mouse meninges imaged by fluorescent stereo microscopy (*n* = 12; bars, 500 μm) **(B)** or confocal imaging (*n* = 3; bars, 100 μm) **(C)**. **(D)** LYVE-1 and PROX-1 immunostaining of meninges (*n* = 4). Bars, 500 μm. **(E–G)** Expression pattern of GFP, LYVE-1 and PECAM adjacent to the venous sinuses of the meninges of *Prox1*^*GFP*^ lymphatic reporter mice at P21. Images are shown for native GFP signal (*n* = 5; bars, 500 μm) **(E)**, GFP and LYVE-1 immunostaining detected by fluorescent stereo microscopy (*n* = 6; bars, 500 μm) **(F)** or confocal microscopy (*n* = 3; bars, 200 μm) **(G)**. **(H)** Expression pattern of GFP and PECAM shown by fluorescent stereo microscopy after whole-mount immunostaining of wild type meninges (*n* = 5). Bars, 500 μm. **(I)** Expression of YFP and LYVE-1 adjacent to the venous sinuses of the meninges of young adult *Flt4*^*YFP*^ lymphatic reporter mice shown by GFP and LYVE-1 immunostaining (*n* = 3). Bars, 500 μm. Representative images are shown in all panels.

First, we visualized the expression pattern of lymphatic markers in the meningeal compartment. LYVE-1, PDPN and PROX-1 positive LECs were detectable along the transverse and sagittal sinuses in young adult (P21) C57BL/6 wild type mice (Figures 1A–D). These structures also carry the PECAM panendothelial marker (Figure 1A). *Prox1*^*GFP*^ and *Flt4*^*YFP*^ lymphatic reporter animals, which express GFP or YFP in all PROX-1 or VEGFR3 positive LECs, show fluorescent signal adjacent to the transverse and sagittal sinuses overlapping with LYVE-1 and PECAM molecules shown by fluorescent stereo microscopy and confocal imaging (Figures 1E–I). These studies confirmed the presence of lymphatics in the dura mater.

### Meningeal Lymphatics Take Up and Transport Macromolecules From the CNS

**Figure 2:**
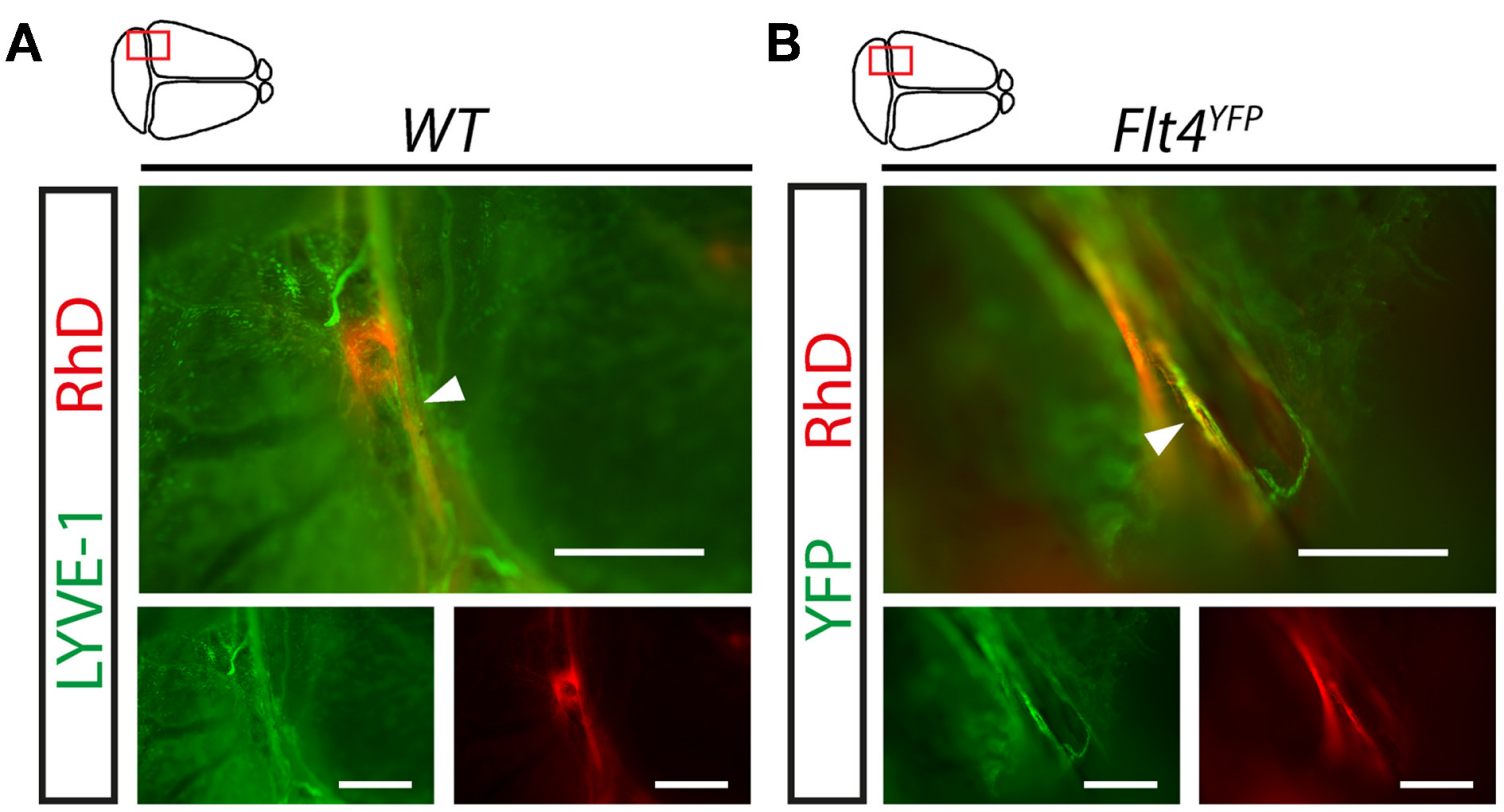
Uptake and transport of fluorescently labeled macromolecules injected into the CNS by the meningeal lymphatic vessels. **(A,B)** Monitoring the uptake and transport of 70 kDa RhD by the meningeal lymphatic vessels shown in whole-mount stained meninges of young adult wild type mice using the LYVE-1 marker **(A)** and YFP signal in *Flt4*^*YFP*^ lymphatic reporter mice **(B)** imaged by fluorescent stereo microscopy after intraparenchymal injection with 70 kDa RhD. Representative images are shown of 3 independent experiments. White arrowheads show RhD in the meningeal lymphatic vessels. Bars, 500 μm.

The contribution of meningeal lymphatic structures to the transport of macromolecules from the CNS is not clear. While the results of one part of the research groups indicate the involvement of meningeal lymphatics in the transport of large molecules from the CNS, other investigators are not so convinced or were not able to demonstrate the role of meningeal lymphatic vessels in the process (6, 7, 10, 11, 20). The widely used approach for monitoring the transport of macromolecules from the CNS is injecting labeled molecules into the brain parenchyma, ventricles, or cisterna magna. Our results indicate that fluorescently labeled 70 kDa RhD is detectable adjacent to the sinuses after intraparenchymal injection in LYVE-1 positive meningeal lymphatic vessels in young adult animals and in *Flt4*^*YFP*^ mice in the VEGFR3 positive lymphatic vessels (Figures 2A,B).

### Meningeal Lymphatics Develop During the Postnatal Period, Which Process Involves Their Structural Maturation

**Figure 3:**
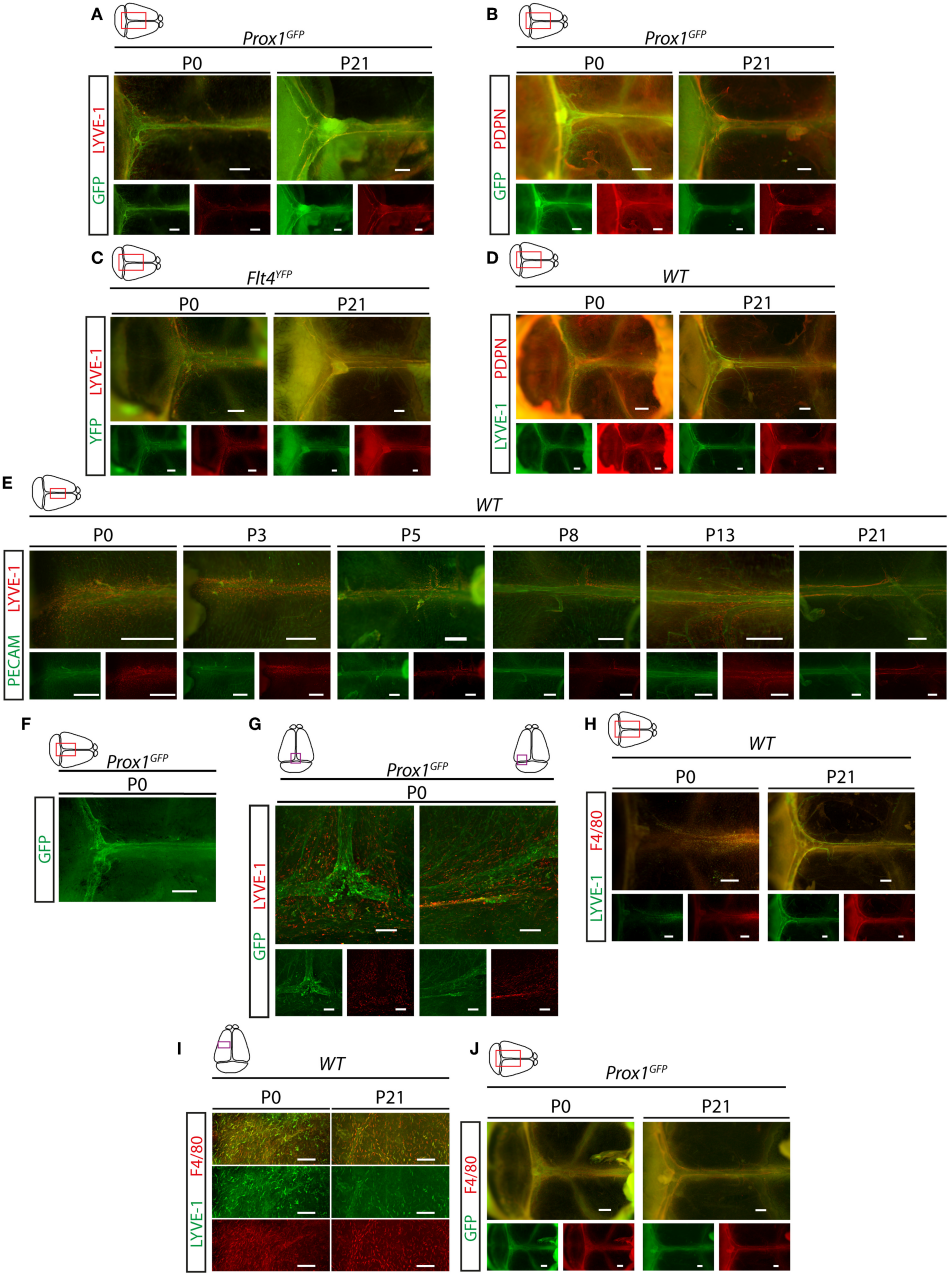
Characterization of the developmental program of meningeal lymphatics. **(A)** Expression of GFP and LYVE-1 adjacent to the venous sinuses of whole-mount immunostained meninges of *Prox1*^*GFP*^ lymphatic reporter mice at P0 and P21 (P0 *n* = 9; P21 *n* = 6). Bars, 500 μm. **(B)** Expression of GFP and PDPN adjacent to the venous sinuses of whole-mount immunostained meninges of *Prox1*^*GFP*^ lymphatic reporter mice at P0 and P21 (P0 *n* = 6; P21 *n* = 6). Bars, 500 μm. **(C)** Expression of YFP and LYVE-1 in the dura mater of *Flt4*^*YFP*^ lymphatic reporter mice detected by whole-mount immunostaining at P0 and P21 (P0 *n* = 6; P21 *n* = 3). Bars, 500 μm. **(D)** LYVE-1 and PDPN expression shown in the meninges of P0 and P21 wild type mice (P0 *n* = 8; P21 *n* = 8). Bars, 500 μm. **(E)** PECAM and LYVE-1 expression adjacent to the superior sagittal sinus at P0, P3, P5, P8, P13 and P21 in wild type mice (P0 *n* = 6; P3 *n* = 5; P5 *n* = 2; P8 *n* = 2; P13 *n* = 3; P21 *n* = 7). Bars, 500 μm. **(F)** GFP signal detected by fluorescent stereo microscopy (*n* = 18) in meninges of *Prox1*^*GFP*^ lymphatic reporter mice at P0. Bars, 500 μm. **(G)** GFP and LYVE-1 expression detected by confocal microscopy after whole-mount immunostaining of meninges of *Prox1*^*GFP*^ lymphatic reporter mice at P0. Bars, 200 μm. **(H,I)** LYVE-1 and F4/80 expression shown in the meninges of wild type mice at P0 and P21 imaged by fluorescent stereo microscopy (P0 *n* = 9; P21 *n* = 6; bars, 500 μm) **(H)** or confocal imaging (bars, 200 μm) **(I)**. **(J)** GFP and F4/80 expression in the meninges of P0 and P21 *Prox1*^*GFP*^ reporter mice (P0 *n* = 3; P21 *n* = 3). Bars, 500 μm. Representative images are shown in all panels.

Next, we characterized the developmental program of meningeal lymphatics. Meningeal lymphatics exhibit a fully mature morphology by P21 along the transverse and sagittal sinuses shown by Prox1-GFP, Flt4-YFP, LYVE-1, PDPN, and PECAM markers (Figures 3A–E). Between P0 and P21 a structural remodeling and maturation process of the primary network occurs as it is shown in *Prox1*^*GFP*^ and *Flt4*^*YFP*^ lymphatic reporter mice and by the immunostaining of lymphatic and panendothelial markers (Figures 3A–G). At P0 a premature structure can be detected expressing Prox1-GFP, Flt4-YFP and PDPN markers, which structure is surrounded by LYVE-1 positive single cells, and the expression of LYVE-1 and F4/80 are detectable in these single cells adjacent to the developing lymphatic network shown by fluorescent stereo microscopy and confocal imaging (Figures 3H–J). The number of these LYVE-1 positive cells is reduced by P21, while several F4/80 positive single cells are still present (Figures 3H–J).

### Macromolecules Injected Into the CNS Are Transported to the Deep Cervical Lymph Nodes

**Figure 4:**
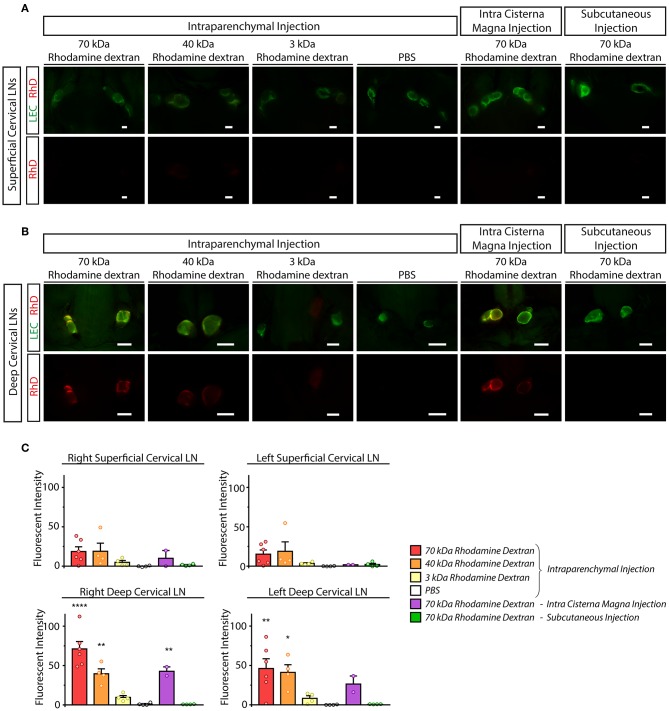
Monitoring the drainage of macromolecules from the CNS to the superficial and deep cervical lymph nodes. **(A,B)** Accumulation of the injected labeled macromolecules in the superficial cervical lymph nodes **(A)** and deep cervical lymph nodes **(B)** of lymphatic reporter mice after intraparenchymal injection of 70, 40 and 3 kDa RhD, PBS, intra cisterna magna injection of 70 kDa RhD and subcutaneous injection of 70 kDa RhD at the same injection site which was used for the intraparenchymal injections (*n* = 6 for 70 kDa RhD intraparenchymal injection; *n* = 4 for 40 kDa RhD intraparenchymal injection; *n* = 4 for 3 kDa RhD intraparenchymal injection; *n* = 4 for PBS intraparenchymal injection; *n* = 2 for 70 kDa intra cisterna magna injection; *n* = 4 for 70 kDa RhD subcutaneous injection). Bars, 1,000 μm. Representative images are shown. **(C)** Quantitative data are shown for mean fluorescent intensity in the lymph nodes of the cervical region (mean fluorescent intensity of background subtracted from the mean fluorescent intensity of lymph nodes) compared to PBS injection (One-way ANOVA, Dunnett's *post-hoc* test; mean ± SEM; *n* = 3 or more animals for each group; *n* = 6 for 70 kDa RhD intraparenchymal injection; *n* = 4 for 40 kDa RhD intraparenchymal injection; *n* = 4 for 3 kDa RhD intraparenchymal injection; *n* = 4 for PBS intraparenchymal injection; *n* = 2 for 70 kDa intra cisterna magna injection *n* = 4 for 70 kDa RhD subcutaneous injection; *P < 0.05 vs. intraparenchymal PBS injection; **P < 0.01 vs. intraparenchymal PBS injection; ****P < 0.0001 vs. intraparenchymal PBS injection).

Next, we monitored the transport of fluorescently labeled macromolecules from the CNS to the cervical lymph nodes in experimental mice. 70, 40, and 3 kDa RhD conjugates were injected into the brain parenchyma and cisterna magna. In parallel, intraparenchymal injection of PBS and subcutaneous injection of 70 kDa RhD (at the same injection site which was used for the intraparenchymal injections) were also performed as controls. Minimal drainage of the fluorescent tracers was detected to the superficial cervical lymph nodes compared to the controls (Figure 4A). In contrast, the transport of the labeled 70 and 40 kDa macromolecules was robust to the deep cervical lymph nodes after intraparenchymal and cisterna magna injections (Figure 4B). The quantification of the drainage also revealed the significant and strong drainage of the fluorescent macromolecules to the deep cervical lymph nodes after parenchymal and cisterna magna injections (Figure 4C). The drainage of macromolecules after intraparenchymal injection showed ipsilateral dominance (Figure 4C). Macromolecule drainage to the cervical area after intraparenchymal (or intra cisterna magna) injections of the fluorescently labeled tracers in live animals was also monitored at various time points (data not shown). The fluorescent macromolecules were detectable in the deep cervical lymph nodes after 30 min, and there was an increase in the fluorescent signal by 120 min in live animals (data not shown). These experiments revealed no difference compared to the studies when the animals were sacrificed just before performing fluorescent microscopy.

### The Structural Remodeling of the Meningeal Lymphatic Vessels Coincides With the Increase of the Transport of Macromolecules to the Deep Cervical Lymph Nodes

**Figure 5:**
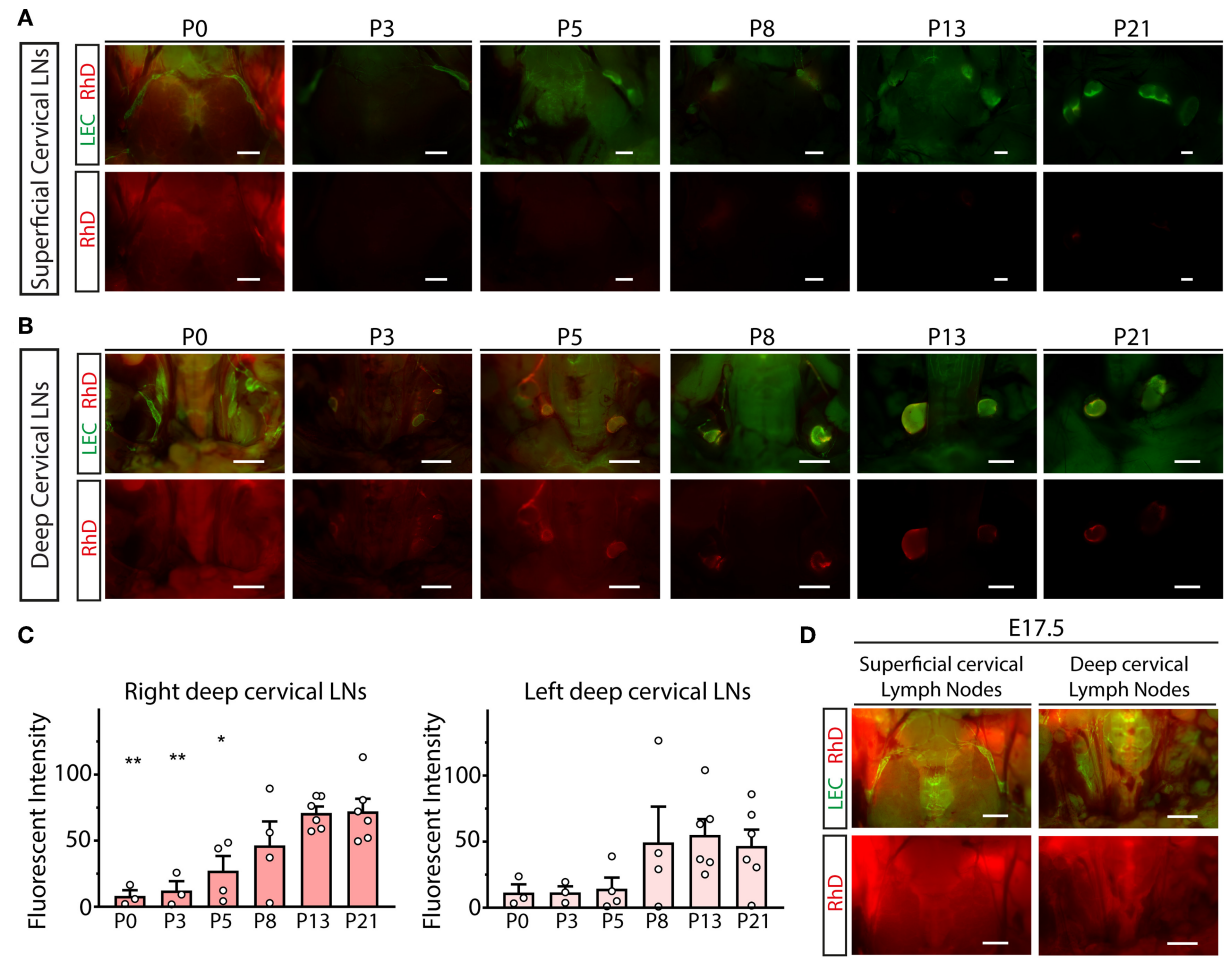
Monitoring the drainage of macromolecules from the CNS during the first three postnatal weeks. **(A,B)** Drainage of 70 kDa RhD into the superficial cervical lymph nodes **(A)** and deep cervical lymph nodes **(B)** of lymphatic reporter mice at P0, P3, P5, P8, P13 and P21 after intraparenchymal injection (P0 *n* = 3; P3 *n* = 3; P5 *n* = 4; P8 *n* = 4; P13 *n* = 6; P21 *n* = 6). Bars, 1,000 μm. **(C)** Quantitative data for mean fluorescent intensity in the deep cervical lymph nodes (mean fluorescent intensity of background subtracted from the mean fluorescent intensity of lymph nodes) after intraparenchymal injection of 70 kDa RhD into lymphatic reporter animals at P0, P3, P5, P8, P13 and P21 (One-way ANOVA, Dunnett's *post-hoc* test; mean ± SEM; *n* = 3 or more animals for each group; P0 *n* = 3; P3 *n* = 3; P5 *n* = 4; P8 *n* = 4; P13 *n* = 6; P21 *n* = 6; *P < 0.05 vs. P21; **P < 0.01 vs. P21). **(D)** Drainage of 70 kDa RhD into the superficial and deep cervical lymph nodes of *Flt4*^*YFP*^ reporter mice at E17.5 after *in utero* intraparenchymal injection (*n* = 2). Bars, 1,000 μm. Representative images are shown.

To characterize the drainage of macromolecules from the CNS to the cervical area, intraparenchymal injection of 70 kDa RhD was performed between P0 and P21. The transport of the labeled macromolecule was robust at P21 to the deep cervical lymph nodes, dominantly to the injection side (Figures 5A–C). In contrast, the drainage was reduced at earlier time points, and almost no drainage was detected at P0 (Figures 5B,C). Quantitative data also showed that the drainage of the labeled macromolecule is increasing between P0 and P21 (Figure 5C). The transport of the labeled macromolecules to the cervical region is also not detectable after the intraparenchymal injection of the E17.5 embryos *in utero* (Figure 5D). These results indicate that the efficient transport of macromolecules to the deep cervical lymph nodes coincides with the maturation of the meningeal lymphatic network (shown in Figure 3).

### Characterization of the Phenotype of *Plcγ*2^−/−^ Embryos and Mice Induced by Backflow of Blood Into the Lymphatic System

**Figure 6:**
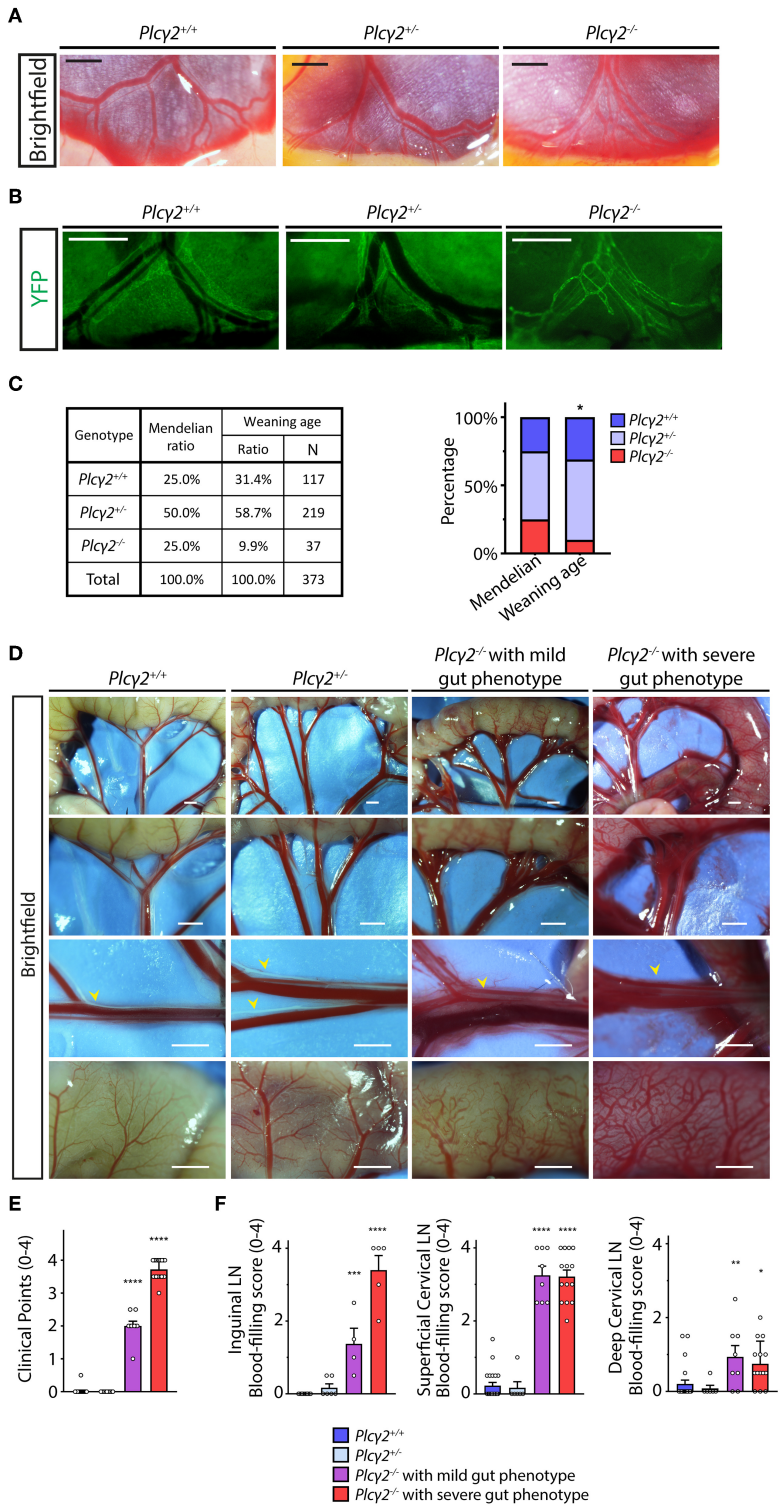
Characterization of the lymphatic phenotype of *Plcγ*2^−/−^ and littermate control mice. **(A)** Lymphatic and blood vessels are shown in the wall of small intestine and mesentery of *Plc*γ*2*^+/+^*, Plc*γ*2*^+/−^ and *Plc*γ*2*^−/−^ embryos at E19.5 (*n* = 6 for *Plc*γ*2*^+/+^ embryos; *n* = 7 for *Plc*γ*2*^+/−^ embryos, *n* = 6 for *Plc*γ*2*^−/−^ embryos). Bars, 500 μm. **(B)** Fluorescent stereo microscopic images are shown of YFP-positive lymphatic vessels of the gut wall and mesentery of *Plc*γ*2*^+/+^*, Plc*γ*2*^+/−^ and *Plc*γ*2*^−/−^ embryos at E19.5 on a *Flt4*^*YFP*^ lymphatic reporter background (*n* = 3 for *Plc*γ*2*^+/+^ embryos; *n* = 3 for *Plc*γ*2*^+/−^ embryos; *n* = 3 for *Plc*γ*2*^−/−^ embryos). Bars, 500 μm. **(C)** Genotype of the offsprings of *Plc*γ*2* colony at weaning age compared to Mendelian distribution. Contingency table and stacked bar graph are shown. Pearson's Chi-square test was performed for testing equality of percentile profiles of allele pairs distribution of 373 offsprings from 67 litters of *Plc*γ*2*^+/−^ × *Plc*γ*2*^+/−^ matings at weaning age compared to Mendelian allele pairs distribution. *P < 0.05. **(D)** Images of lymphatic and blood vessels in the small intestine and mesentery are shown of young adult *Plc*γ*2*^+/+^*, Plc*γ*2*^+/−^ and *Plc*γ*2*^−/−^ mice with mild or severe gut phenotype (*n* = 26 for *Plc*γ*2*^+/+^, *n* = 9 for *Plc*γ*2*^+/−^, *n* = 9 for *Plc*γ*2*^−/−^ mice with mild gut phenotype, *n* = 18 for *Plc*γ*2*^−/−^ mice with severe gut phenotype). Yellow arrowheads point to mesenteric lymphatic vessels. Bars, 1,000 μm. All images are representative. **(E)** Quantitative data are shown for clinical scores for blood-filling and malformations of lymphatics in the mesentery and small intestine of *Plc*γ*2*^+/+^*, Plc*γ*2*^+/−^ and *Plc*γ*2*^−/−^ mice with mild or severe gut phenotype (Mean ± SEM; One-way ANOVA; Dunnett's *post-hoc* test; *n* = 26 for *Plc*γ*2*^+/+^, *n* = 9 for *Plc*γ*2*^+/−^, *n* = 9 for *Plc*γ*2*^−/−^ mice with mild gut phenotype, *n* = 18 for *Plc*γ*2*^−/−^ mice with severe gut phenotype; ****P < 0.0001 vs. *Plc*γ*2*^+/+^). **(F)** Clinical scores for presence of blood in lymph nodes of inguinal lymph nodes, superficial cervical lymph nodes and deep cervical lymph nodes of young adult *Plc*γ*2*^+/+^*, Plc*γ*2*^+/−^ and *Plc*γ*2*^−/−^ mice with mild or severe gut phenotype are shown (0 point: no blood filling of lymph node detected; 4 points: severe blood filling of lymph node; One-way ANOVA, Dunnett's *post-hoc* test; mean ± SEM; *n* = 2 or more animals for each group; Inguinal lymph nodes: *n* = 6 mice for *Plc*γ*2*^+/+^, *n* = 3 mice for *Plc*γ*2*^+/−^, *n* = 2 mice for *Plc*γ*2*^−/−^ mice with mild gut phenotype, *n* = 3 mice for *Plc*γ*2*^−/−^ mice with severe gut phenotype; Cervical lymph nodes: *n* = 11 mice for *Plc*γ*2*^+/+^, *n* = 3 mice for *Plc*γ*2*^+/−^, *n* = 4 mice for *Plc*γ*2*^−/−^ mice with mild gut phenotype, *n* = 7 mice for *Plc*γ*2*^−/−^ mice with severe gut phenotype; *P < 0.05 vs. *Plc*γ*2*^+/+^*;* **P < 0.01 vs. *Plc*γ*2*^+/+^*;* ***P < 0.001 vs. *Plc*γ*2*^+/+^*;* ****P < 0.0001 vs. *Plc*γ*2*^+/+^). Representative images are shown.

We aimed to characterize the role of the mechanical forces generated by lymph flow as possible regulators of the developmental program of meningeal lymphatics. It is known that mouse models lacking the components of the CLEC2, SYK, SLP76, PLCγ2 signaling pathway in platelets develop backflow of blood from the venous system into the lymphatics (31–33, 35). This phenotype is present because of the loss of platelet activation by LECs at the lympho-venous junction, where the thoracic duct meets the subclavian vein. In prior reports CLEC2-deficient mice were used to define the role of lymphatic function in the gut and lung (25, 36, 37). To characterize the phenotype of PLCγ2-deficient embryos, the strain was crossed to the *Flt4*^*YFP*^ lymphatic reporter background. The mesenteric lymphatic vessels are blood-filled and a premature network of mesenteric lymphatic vessels is present in *Plcγ*2^−/−^ embryos during late gestation compared to *Plcγ*2^+/+^ and *Plcγ*2^+/−^ littermates, indicating the defective structural maturation of mesenteric lymphatic vessels (Figures 6A,B), which process is lymph flow-dependent and also detectable in the absence of CLEC2 (25, 36). These findings suggest that the lymph flow is reduced in PLCγ2-deficient embryos similarly to CLEC2-deficient model.

Next, we defined the survival rate of PLCγ2-deficient mice at weaning age. Only 9.9% of the knockouts (the Mendelian expected ratio is 25 %) were alive at this age (Figure 6C), most likely because of the impairment of pulmonary lymphatic function in the embryonic lung (41). As expected, blood-filled lymphatics were present in the intestinal wall and mesentery of surviving *Plc*γ*2*^−/−^ animals compared to *Plc*γ*2*^+/+^ and *Plc*γ*2*^+/−^ littermate control mice at P21 (Figure 6D). In these experiments *Plc*γ*2*^−/−^ mice with severe or less severe (mild) gut phenotype were detected (Figure 6D) similarly to the CLEC2-deficient and SLP76-deficient models as described before (32, 42). *Plc*γ*2*^−/−^ animals showing mild phenotype at P21 have less blood-filled lymphatic vessels in the mesentery and gut wall compared to the *Plc*γ*2*^−/−^ animals displaying severe gut phenotype (Figure 6D). Thereafter, the severity of the phenotype was assessed in *Plc*γ*2*^−/−^, *Plc*γ*2*^+/+^ and *Plc*γ*2*^+/−^ littermate control mice using a score system, and we set up two separate groups of *Plc*γ*2*^−/−^mice displaying severe or mild gut phenotype (Figure 6E).

We also characterized the structure of the lymph nodes in *Plc*γ*2*^+/+^, *Plc*γ*2*^+/−^ and *Plc*γ*2*^−/−^ littermates (Figure 6F and Supplementary Figure 2). The mesenteric, inguinal and superficial cervical lymph nodes are blood-filled in the model, which is caused by the backflow of the blood (Supplementary Figure 2) as it was reported by others in CLEC2-deficient mice (31, 32). It is also important to note that the deep cervical lymph nodes are not or minimally blood-filled in *Plc*γ*2*^−/−^ mice (Figure 6F and Supplementary Figure 2). This is similar to the phenotype of the lymph nodes of the lung which are also not blood-filled in CLEC2-deficient animals, but the lymphatic function is still greatly reduced in them (37). It is not known why backflow of blood is more complete in the intestine than in the lung or deep cervical lymph nodes, but the fact that the deep cervical lymph nodes in PLCγ2-deficient animals contain no or minimal blood makes the model even better. It is important to appreciate the lymph nodes and vessels without blood in their lumen still experience the reduced lymphatic flow because they feed into more proximal vessels that are obstructed by the backflow of blood from the venous system [as it was shown in (32)]. Moreover, our studies indicate that the basic structure of lymph nodes in *Plc*γ*2*^−/−^ mice was not altered (Supplementary Figure 2B).

### Reduced Lymphatic Function and Lymph Flow in *Plcγ*2^−/−^ Mice

**Figure 7:**
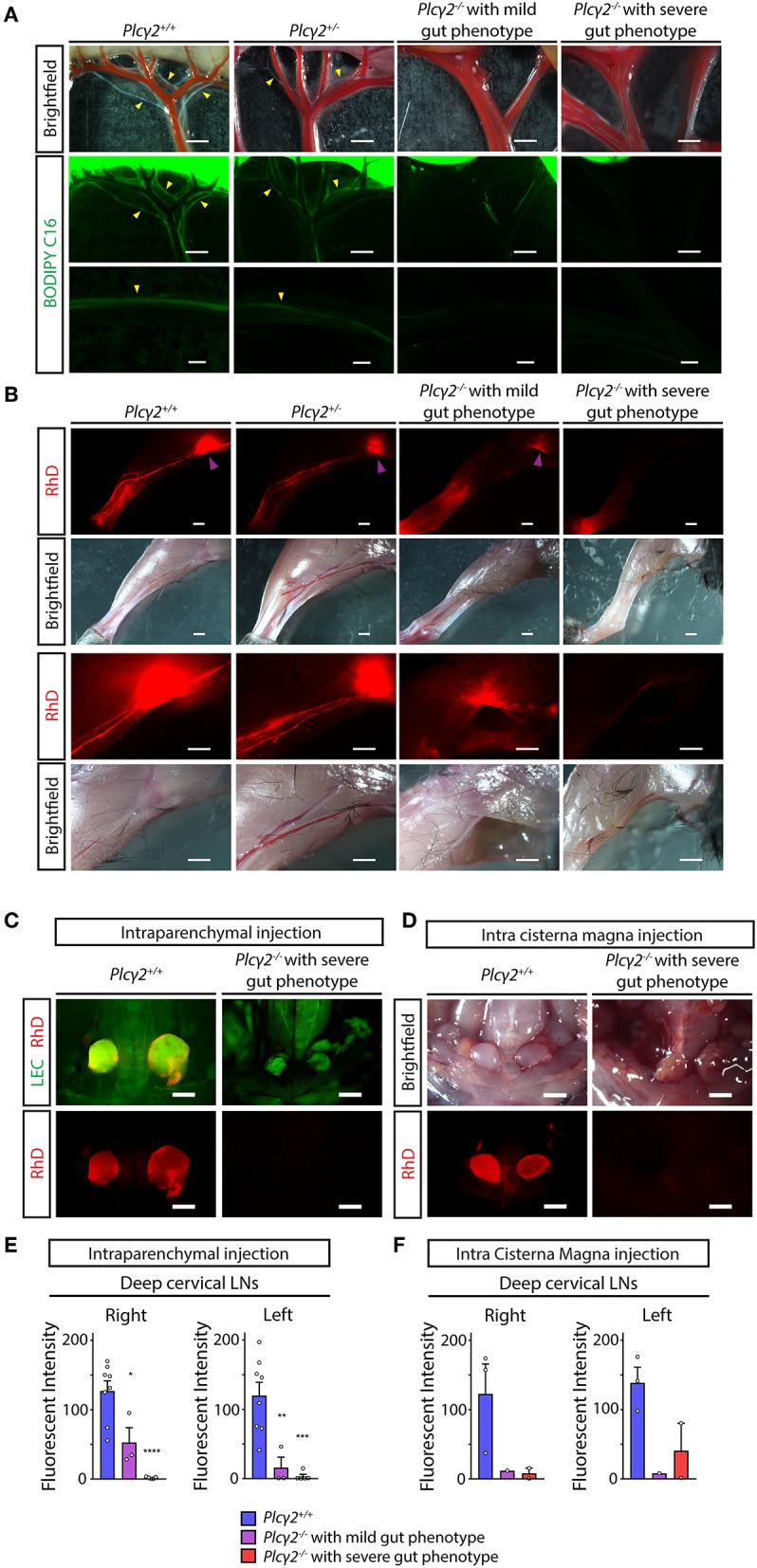
Characterization of lymphatic function in *Plcγ*2^−/−^ and littermate control mice. **(A)** Representative images are shown for uptake and drainage of BODIPY C_16_ by mesenteric lymphatic vessels of young adult *Plc*γ*2*^+/+^*, Plc*γ*2*^+/−^ and *Plc*γ*2*^−/−^ mice with mild or severe gut phenotype 2 h after feeding with BODIPY C_16_. Yellow arrowheads point to lymphatic vessels draining BODIPY C_16_ (*n* = 6 mice for *Plc*γ*2*^+/+^, *n* = 3 mice for *Plc*γ*2*^+/−^, *n* = 2 mice for *Plc*γ*2*^−/−^ mice with mild gut phenotype, *n* = 2 mice for *Plc*γ*2*^−/−^ mice with severe phenotype). **(B)** Representative images are shown for uptake and drainage of 70 kDa RhD 100 min after subcutaneous injection to hind limb paws of young adult *Plc*γ*2*^+/+^*, Plc*γ*2*^+/−^ and *Plc*γ*2*^−/−^ mice with mild or severe gut phenotype. Purple arrowheads show RhD signal in popliteal lymph nodes (*n* = 6 mice for *Plc*γ*2*^+/+^, *n* = 3 mice for *Plc*γ*2*^+/−^, *n* = 2 mice for *Plc*γ*2*^−/−^ mice with mild gut phenotype, *n* = 2 mice for *Plc*γ*2*^−/−^ mice with severe gut phenotype). **(C,D)** Images shown for drainage of 70 kDa RhD into the deep cervical lymph nodes after intraparenchymal injection of the fluorescently labeled macromolecule to *Plc*γ*2*^+/+^ and *Plc*γ*2*^−/−^ mice with severe gut phenotype on a *Flt4*^*YFP*^ lymphatic reporter background **(C)** or injection of the tracer into cisterna magna of *Plc*γ*2*^+/+^ and *Plc*γ*2*^−/−^ mice with severe gut phenotype **(D)** (*n* = 5 for intraparenchymal injection; *n* = 2 for intra cisterna magna injection). Bars, 1,000 μm. All images are representative. **(E)** Quantitative data are shown for mean fluorescent intensity in the deep cervical lymph nodes (mean fluorescent intensity of background subtracted from the mean fluorescent intensity of lymph nodes) after intraparenchymal injection of 70 kDa RhD into *Plc*γ*2*^+/+^ and *Plc*γ*2*^−/−^ mice with severe or mild phenotype in the gut (Mean ± SEM; One-way ANOVA; Dunnett's *post-hoc* test; *n* = 8 for *Plc*γ*2*^+/+^ mice; *n* = 3 for *Plc*γ*2*^−/−^ mice with mild gut phenotype, *n* = 5 for *Plc*γ*2*^−/−^ mice with severe gut phenotype; *P < 0.05 vs. *Plc*γ*2*^+/+^*;* **P < 0.01 vs. *Plc*γ*2*^+/+^*;* ***P < 0.001 vs. *Plc*γ*2*^+/+^*;* ****P < 0.0001 vs. *Plc*γ*2*^+/+^). **(F)** Quantitative data are shown for drainage to the deep cervical lymph nodes (mean fluorescent intensity of background subtracted from the mean fluorescent intensity of lymph nodes) after intra cisterna magna injection of 70 kDa RhD into *Plc*γ*2*^+/+^ and *Plc*γ*2*^−/−^ mice with severe or mild phenotype in the gut (Mean ± SEM; One-way ANOVA; Dunnett's *post-hoc* test; *n* = 3 for *Plc*γ*2*^+/+^ mice; *n* = 1 for *Plc*γ*2*^−/−^ mice with mild gut phenotype, *n* = 2 for *Plc*γ*2*^−/−^ mice with severe gut phenotype).

Next, we characterized the lymphatic function in various organs in PLCγ2-deficient mice. Lymphatic function and lymph flow are critical in lipid absorption from the small intestine. First, fluorescently labeled lipid (BODIPY C_16_) was used to feed PLCγ2-deficient mice to assess the lymphatic function in the gut. As it is shown in Figure 7A normal transport of the fluorescently labeled lipid was visualized in *Plc*γ*2*^+/+^ and *Plc*γ*2*^+/−^ mice, but there was no detectable lymphatic function in *Plc*γ*2*^−/−^ mice with severe gut phenotype. Please note that the transport of the fluorescently labeled lipid was less reduced in *Plc*γ*2*^−/−^ mice with mild gut phenotype.

Second, as a large molecule 70 kDa RhD was injected into the hind paw, and the lymph flow of the hind limb was monitored and the drainage to the lymph nodes was assessed (Figure 7B). The lymphatic function was greatly impaired in *Plc*γ*2*^−/−^ mice with severe gut phenotype, and it was less reduced in *Plc*γ*2*^−/−^ mice with mild gut phenotype (Figure 7B).

Third, the drainage of labeled macromolecules from the CNS after intraparenchymal or intra cisterna magna injection was assessed in *Plc*γ*2*^+/+^ and *Plc*γ*2*^−/−^ littermates. While the transport to the deep cervical lymph nodes in *Plc*γ*2*^+/+^ mice was significant both after intraparenchymal and intra cisterna magna injections, it was greatly reduced in *Plc*γ*2*^−/−^ mice with severe gut phenotype (Figures 7C,D). The quantification of the data also indicates significant drainage to the deep cervical lymph nodes in control mice, and reduced transport in *Plc*γ*2*^−/−^ littermates with severe gut phenotype (Figures 7E,F). Of note, the drainage of the labeled macromolecules was less reduced to the deep cervical lymph nodes in *Plc*γ*2*^−/−^ mice displaying less severe phenotype in the mesentery and small intestine after intraparenchymal and intra cisterna magna injections (Figures 7E,F). Collectively, these findings indicate that the reduction of the lymphatic function and lymph flow correlates with the gut phenotype of the animals. If the gut phenotype is less severe, the lymph flow is less reduced. We believe that similarly to the previous reports using the CLEC2-deficient strain referenced in (25, 36, 37), we have established an excellent model to study the possible role of lymph flow in *in vivo* experiments.

### Lymph Flow-Induced Structural Maturation and Remodeling of the Meningeal Lymphatic Vessels

**Figure 8:**
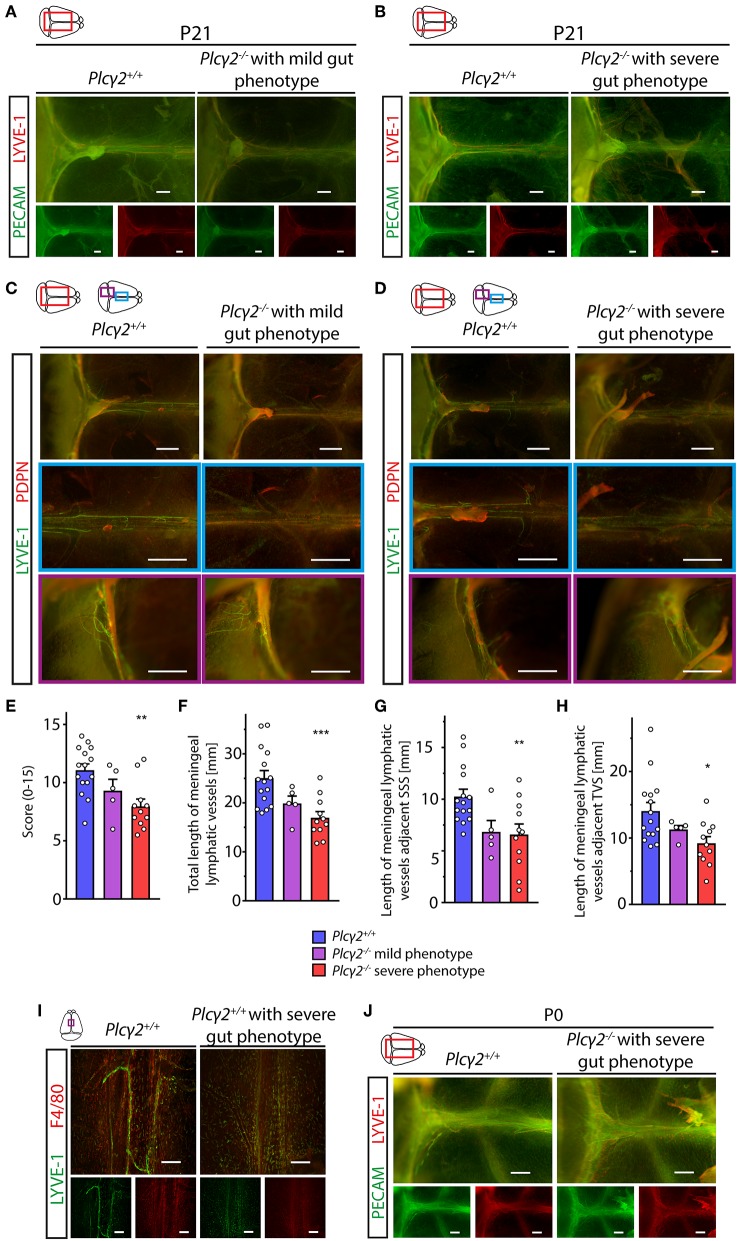
Morphological characterization of the meningeal lymphatics in *Plc*γ*2*^+/+^ and *Plc*γ*2*^−/−^ mice. **(A,B)** Images are shown of PECAM and LYVE-1 expression in the meninges of young adult *Plc*γ*2*^−/^^−^ and *Plc*γ*2*^+/+^ mice with mild **(A)** or severe **(B)** gut phenotype (*n* = 8 for *Plc*γ*2*^+/+^ mice; *n* = 3 for *Plc*γ*2*^−/^^−^ mice with mild gut phenotype; *n* = 4 for *Plc*γ*2*^−/^^−^ mice with severe gut phenotype). Bars, 500 μm. **(C,D)** Images are shown of LYVE-1 and PDPN expression next to the transverse sinus and superior sagittal sinus of *Plc*γ*2*^+/+^ (*n* = 8), *Plc*γ*2*^−/−^ mice with mild (*n* = 3) **(C)** and severe (*n* = 6) **(D)** gut phenotype. Bars, 1,000 μm. **(E)** Quantitative data are shown for clinical scores (0–15) representing structural malformations of the meningeal lymphatics in *Plc*γ*2*^+/+^ and *Plc*γ*2*^−/−^ mice with severe or mild phenotype in the gut (Mean ± SEM; One-way ANOVA; Dunnett's *post-hoc* test; *n* = 15 for *Plc*γ*2*^+/+^ mice; *n* = 5 for *Plc*γ*2*^−/−^ mice showing mild gut phenotype, *n* = 11 for *Plc*γ*2*^−/−^ mice displaying severe gut phenotype; **P < 0.01 vs. *Plc*γ*2*^+/+^). **(F–H)** Quantitative data are shown for differences in the total length of the meningeal lymphatics **(F)**, total length of the meningeal lymphatics adjacent to the superior sagittal sinus **(G)**, and adjacent to the transverse sinuses **(H)** in *Plc*γ*2*^+/+^ and *Plc*γ*2*^−/−^ mice displaying severe or mild phenotype in the gut (Mean ± SEM; One-way ANOVA; Dunnett's *post-hoc* test; *n* = 15 for *Plc*γ*2*^+/+^ mice; *n* = 5 for *Plc*γ*2*^−/−^ mice showing mild gut phenotype, *n* = 11 for *Plc*γ*2*^−/−^ mice displaying severe gut phenotype; *P < 0.05 vs. *Plc*γ*2*^+/+^*;* **P < 0.01 vs. *Plc*γ*2*^+/+^*;* ***P < 0.001 vs. *Plc*γ*2*^+/+^). **(I)** Confocal images are shown of LYVE-1 and F4/80 immunostaining of whole-mount meninges of young adult *Plc*γ*2*^+/+^ and *Plc*γ*2*^−/−^ mice with a severe gut phenotype (*n* = 3 for each group). Bars, 200 μm. **(J)** Images are shown of PECAM and LYVE-1 expression in the meninges of *Plc*γ*2*^+/+^ and *Plc*γ*2*^−/^^−^ mice with severe gut phenotype at P0 (*n* = 5 for both groups). Bars, 500 μm. All images are representative.

Next, we characterized the morphology of the meningeal lymphatics in animals with reduced lymphatic function and lymph flow. Surprisingly, the structural development of the meningeal lymphatic vessels was impaired in *Plc*γ*2*^−/−^ mice displaying severe gut phenotype shown by immunostaining of LYVE-1-PECAM and LYVE-1-PDPN markers at P21 (Figures 8A–D). The defect in the structural maturation of meningeal lymphatics was less severe in the *Plc*γ*2*^−/−^ mice showing mild gut phenotype at P21 (Figures 8A,C), but LYVE-1-PECAM and LYVE-1-PDPN staining of lymphatic markers revealed a dramatic impairment of the maturation process in *Plc*γ*2*^−/−^ mice displaying severe gut phenotype at P21 (Figures 8B,D). The evaluation of the images using a score system for the maturation process and length measurements of meningeal lymphatic vessels also revealed the correlation between the impaired maturation process of meningeal lymphatics and the severity of the altered lymphatic function and gut phenotype in the genetic model (Figures 8E–H). The defect of the structural maturation of meningeal lymphatics was also demonstrated by confocal imaging using LYVE-1 immunostaining (Figure 8I). Please note the presence of several LYVE-1 positive single cells next to the superior sagittal sinus in *Plc*γ*2*^−/−^ mice displaying severe gut phenotype but not in *Plc*γ^+/+^ mice at P21 (Figure 8I). Of note, at P0 there is no significant difference in the expression pattern of the markers immunostained with PECAM and LYVE-1 markers in *Plc*γ*2*^−/−^ mice (Figure 8J). Collectively, these findings indicate that lymph flow may act as an important regulator of the maturation and structural remodeling of meningeal lymphatics.

### Defective Maturation of Meningeal Lymphatics Affects the Uptake and Transport of Macromolecules From the CNS

**Figure 9:**
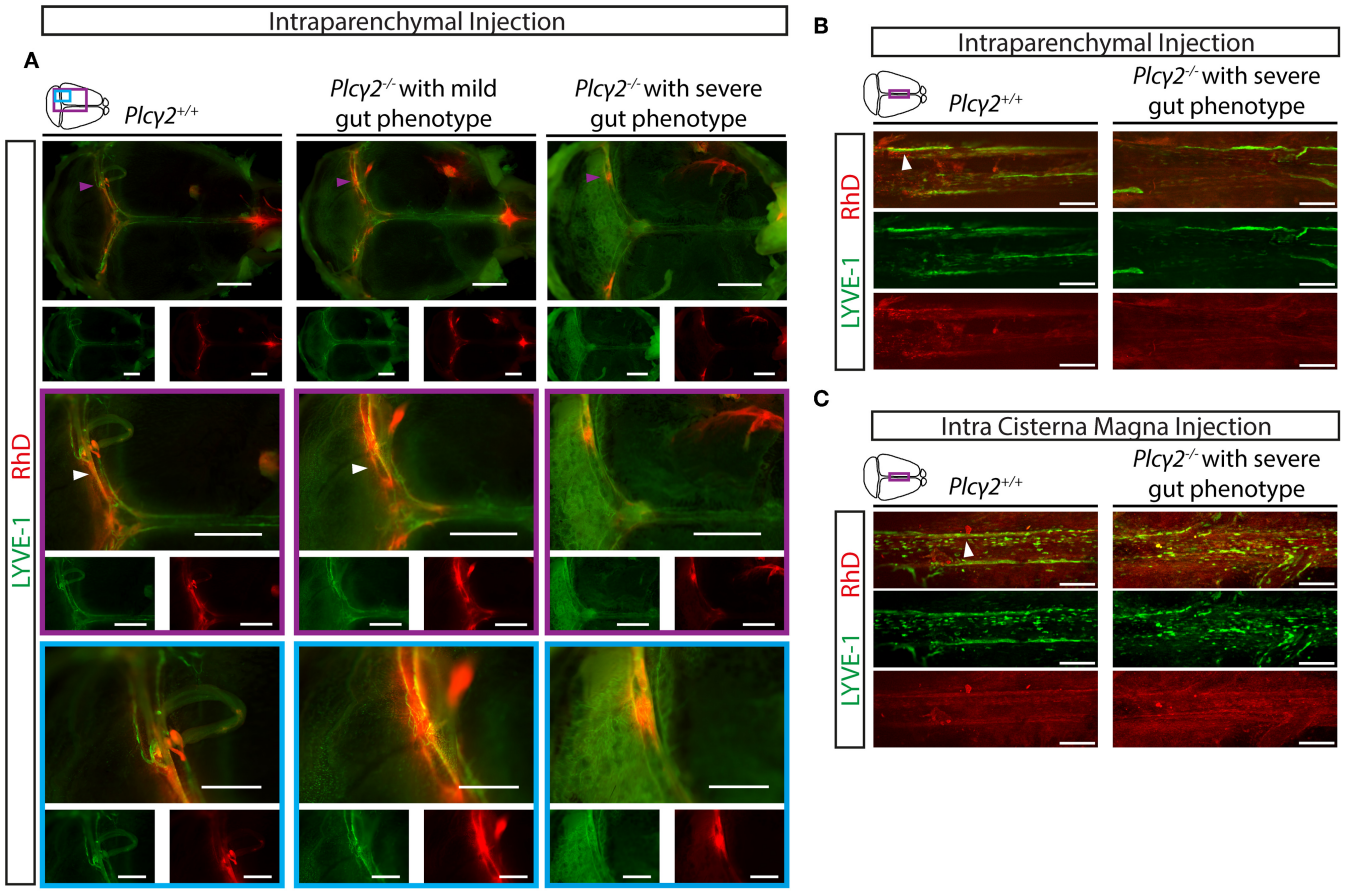
Uptake and transport of labeled macromolecules by the meningeal lymphatics in *Plcγ*2^+/+^ and *Plcγ*2^−/−^ mice. **(A)** Uptake and transport of 70 kDa RhD by the meningeal lymphatic vessels after intraparenchymal injection of young adult *Plc*γ*2*^+/+^, *Plc*γ*2*^−/−^ mice displaying mild or severe gut phenotype. Representative images are shown of whole-mount anti-LYVE-1 immunostaining of meninges of young adult mice (*n* = 8 for *Plc*γ*2*^+/+^ mice; *n* = 3 for *Plc*γ*2*^−/−^ mice showing mild gut phenotype, *n* = 5 for *Plc*γ*2*^−/−^ mice displaying severe gut phenotype). Purple arrowheads point to spots with a high intensity for macromolecule uptake, white arrowheads show meningeal lymphatic vessels draining RhD. Bars, 1,000 μm. **(B,C)** Confocal images are shown of region adjacent to the superior sagittal sinus of whole-mount immunostained meninges of young adult *Plc*γ*2*^+/+^ and *Plc*γ*2*^−/−^ mice after intraparenchymal **(B)** or intra cisterna magna **(C)** injection of 70 kDa RhD (intraparenchymal injection: *n* = 3 for *Plc*γ*2*^+/+^ mice; *n* = 3 for *Plc*γ*2*^−/−^ mice displaying severe gut phenotype; intra cisterna magna injection: *n* = 3 for *Plc*γ*2*^+/+^ mice; *n* = 2 for *Plc*γ*2*^−/−^ mice displaying severe gut phenotype). White arrowheads show meningeal lymphatic vessels draining RhD. Bars, 200 μm.

Thereafter, the uptake of macromolecules into the meningeal lymphatic vessels was characterized in *Plc*γ*2*^+/+^ and *Plc*γ*2*^−/−^ littermate mice. The uptake and transport of labeled macromolecules were detected into the meningeal lymphatic vessels in *Plc*γ*2*^+/+^ mice at P21 after intraparenchymal injection (Figure 9A), similarly as it was shown in Figure 2. In contrast, the process was impaired in *Plc*γ*2*^−/−^ mice displaying severe gut phenotype (Figure 9A). Moreover, the uptake of the labeled macromolecule was less impaired in *Plc*γ*2*^−/−^ mice displaying mild gut phenotype (Figure 9A). Of note, the highly intense spots near the transverse sinus are also present not only in the *Plc*γ*2*^+/+^ but also in the *Plc*γ*2*^−/−^ animals (Figure 9A). Confocal imaging also revealed normal uptake and transport of fluorescently labeled macromolecules in *Plc*γ*2*^+/+^ mice after intraparenchymal or intra cisterna magna injections, while the structure of meningeal lymphatics was greatly impaired and the macromolecule transport was not present in *Plc*γ*2*^−/−^ mice displaying severe gut phenotype (Figures 9B,C).

## Discussion

We demonstrated the presence of LYVE-1, PROX-1, VEGFR3, PDPN and PECAM positive lymphatic structures in the dura mater (Figure 1), which results are in accordance with former reports (6, 7, 10, 11, 21). We also described that meningeal lymphatics develop during the postnatal period (Figure 3) as it was reported by others (21, 22). However, we show the presence of a premature network surrounded by single cells, which might be a lymphangiogenic LYVE-1 positive cell population that may contribute and help the maturation process of the meningeal lymphatic network (Figure 3). The presence and possible role of a single cell population during the process of lymphangiogenesis was also reported by others, but further detailed studies will be needed to understand how they are involved in this process (21, 22).

Our results indicate that meningeal lymphatic structures are involved in the uptake and transport of macromolecules from the CNS (Figures 2, 9). While one part of the prior reports (mostly published by the Kipnis group) indicate that meningeal lymphatics participate in the transport of large molecules from the CNS, other studies were not able to detect the transport of macromolecules by meningeal lymphatics and indicated that further studies are needed to define their role in macromolecule and leukocyte transport (6, 7, 10, 11, 20). Our results support the concept of that meningeal lymphatics are likely players in the transport of macromolecules from the CNS, providing an independent confirmation of the uptake. After our submission another paper has been published in which the authors performed intraparenchymal and intra cisterna magna infusions of the labeled macromolecules, and also detected the uptake and drainage by the meningeal lymphatics (19). Most of the prior studies have been performed after euthanasia of animals, because monitoring the macromolecule transport by the meningeal lymphatics in the intact skull is technically challenging and may have limitations, which prevent the effective detection of the uptake and transport by the meningeal lymphatic structures. In connection, Ma et al. using an elegant approach demonstrated the drainage of macromolecules injected into the CNS to the deep cervical lymph nodes via several different transport routes including the paravascular space of the pia mater and perineural routes in live animals, but the involvement of meningeal lymphatics was unclear based on their work (20), which is in contrast to the findings of others (6, 10). As another aspect, a recent paper by Ma et al. indicated the backflow of the labeled tracer from the CSF to the brain after sacrificing the animals (but not in live mice), which was an unexpected finding because it contradicts the current view (43). Based on this study it is possible that sudden loss of systemic blood pressure after euthanasia may change the macromolecule and fluid movements between the vasculature and the interstitium, which may also influence the detection of the uptake of macromolecules by the meningeal lymphatics. Of note, the uptake of macromolecules into the meningeal lymphatics after euthanasia is not mentioned in that study (43). All the approaches discussed above have limitations because all require the injection of a labeled tracer molecule. An important step forward would be for the whole field to develop a model (e.g., a transgenic mouse) in which the labeled macromolecule is released and secreted directly into the CNS, therefore, the model would not require the injection of the labeled tracer.

The paravascular flow of macromolecules in the neural tissue of the brain is mediated by the glymphatic system (13). However, it is an important question how the meningeal lymphatic vessels are connected to the glymphatic system of the CNS. Our findings in accordance with other reports suggest that some dedicated, special locations close to the transverse sinus are crucial for the formation of the connection between the separate compartments (Figures 2, 9). In recent publications very similar locations were called as “hot spots” by others (10, 19). Further studies using special *in vivo* imaging techniques will be needed to determine the possible mechanisms how these spots connect the meningeal lymphatics to the CNS.

Our results revealed that the drainage of macromolecules injected into the CNS is mediated to the deep cervical lymph nodes after intraparenchymal or intra cisterna magna injections (Figure 4). This result was expected based on the former published works, the drainage of fluorescent tracers from the brain parenchyma has been extensively studied (6, 7, 15–17, 44). Similarly to us, others also detected the draining of macromolecules to both deep cervical lymph nodes (or retropharyngeal lymph nodes) after intraparenchymal injection (or intracortical injection) in adult experimental animals. In these reports a marginal ipsilateral preference of the drainage was described, which difference was not always reported as significant (7, 44, 45). It is important to note that in addition to the drainage of large molecules by the meningeal lymphatics other possible routes of this drainage process are known including the paravascular spaces of the pia mater and other perineural routes (e.g., next to the optic nerve) (20). Further studies are needed to determine the relative contribution of the meningeal lymphatics and the other possible routes to the drainage of macromolecules from the CNS. It is also not known how the meningeal lymphatics exit the skull and how they are connected to the cervical region. A recent report demonstrated that meningeal lymphatics at the skull base are important to mediate the connection (19). It is also possible that the meningeal lymphatics anastomose with other drainage routes.

Importantly, in this study we compared the transport of the labeled macromolecules from the CNS to the maturation process of the meningeal lymphatics (Figure 5). Our results indicate a close correlation between the two processes suggesting that structural maturation of the developing lymphatic network might be an important mechanism to mediate and maintain the effective drainage of macromolecules from the brain.

It has been defined that mechanical forces and shear stress generated by lymph flow are critical regulators of lymphatic endothelial cell gene expression, lymphatic growth and the maturation of the lymphatic network *in vitro* and *in vivo* (24–30). It is well-accepted that in the mouse models lacking the components of the CLEC2, SYK, SLP76 signaling axis in platelets there is backflow of blood from the venous system into the lymphatic system (31–33). This phenotype develops because of the loss of platelet activation by lymphatic endothelial cells at the lympho-venous junction, where the thoracic duct meets the subclavian vein. Studies using the CLEC2-deficient genetic model in which the lymph flow is reduced due to the backflow of blood into the lymphatic system indicated that lymph flow is an essential mechanical regulator of the maturation and remodeling of a premature lymphatic network during the developmental process in the mesentery of the gut (25, 36). In another recent report CLEC2-deficient mice with reduced lymphatic function were used to define the role of pulmonary lymphatics in the postnatal lung (37). PLCγ2 is a component of the same CLEC2, SYK, SLP76 signaling axis with similar phenotype (blood-filled lymphatics in embryos etc.) (34). Here we have performed the detailed analysis of the lymph flow in several organs of the PLCγ2-deficient mice. Our studies revealed reduced lymphatic function in the small intestine and hind limb in PLCγ2-deficient mice (Figures 7A,B). In addition, impairment of the drainage of labeled macromolecules was detected from the CNS after intraparenchymal and intra cisterna magna injections to the deep cervical lymph nodes in *Plc*γ*2*^−/−^ mice (Figures 7C,D). Our results indicate that the lymphatic function is greatly reduced in the hind limb, small intestine and cervical lymph nodes connected to the CNS in *Plc*γ*2*^−/−^ mice. Moreover, the reduction of the flow correlates with the gut phenotype of the animals (Figures 6, 7), indicating that if the gut phenotype is less severe, the lymph flow is less reduced. This is in accordance with former studies, which reported that the backflow of blood can be less severe in some animals, and the phenotype correlates with the impairment of lymph flow (32, 42). Collectively, we believe that we have established a good model to study the possible role of lymph flow in *in vivo* experiments similarly to the previous reports using the CLEC2-deficient strain (25, 36, 37).

Using PLCγ2-deficient mouse strain displaying reduced lymphatic function in various organs we revealed that the remodeling and maturation of meningeal lymphatic structures that occurs during the postnatal period is flow-mediated and flow-dependent (Figure 8). It should be noted that the defect in this maturation process is less severe in animals showing mild gut phenotype, which result further supports the importance of the increasing lymph flow during the development and maturation of the structures (Figure 8). Moreover, the uptake and transport of the labeled macromolecules by the meningeal lymphatics were also affected in animals with impaired lymph flow, while high intensity spots [referred as “hot spots” by others (10)] were still visible in *Plc*γ*2*^−/−^ mice (Figure 9). These findings support that these high intensity spots might be key sites where the connection between the meningeal lymphatics and brain is formed, but to understand the detailed mechanism further studies will be needed. In addition, the defect in the transportation of macromolecules by the meningeal lymphatics was less severe in *Plc*γ*2*^−/−^ animals showing mild gut phenotype (Figure 8C), which finding further supports our conclusions.

Taken together, our studies indicate that meningeal lymphatics are involved in the transport of macromolecules from the CNS. Importantly, lymph flow induced mechanical forces are required for the postnatal formation of mature and functional meningeal lymphatic vessels, which process coincides with increase of drainage of macromolecules from the CNS. Our studies revealing the lymph flow mediated regulation of the development and function of meningeal lymphatics may lead to better understanding of the pathogenesis of neurological diseases such as Alzheimer's disease and neuroinflammatory diseases including multiple sclerosis.

## Data Availability Statement

All datasets generated for this study are included in the article/Supplementary Material.

## Ethics Statement

All animal experiments were approved by the Animal Experimentation Review Board of the Semmelweis University and the Government Office for Pest County (Hungary).

## Author Contributions

LB and ZJ designed the work, interpreted the results, and wrote the paper. LB, ZO, BD, PA, and ZJ performed the experiments and analyzed the data. ZJ supervised the project.

### Conflict of Interest

The authors declare that the research was conducted in the absence of any commercial or financial relationships that could be construed as a potential conflict of interest.

## Supplementary Material

Supplementary Figure 1 Representative images of control immunostainings of meninges. Whole-mount immunostaining and control immunostaining of samples for LYVE-1, PDPN, PROX-1, PECAM, GFP and F4/80 in the dura mater of wild type, *Prox1*^*GFP*^ and *Flt4*^*YFP*^ mice. **(A,B)** Representative images are shown for PECAM and LYVE-1 immunostaining **(A)** and LYVE-1 and PROX-1 immunostaining **(B)** of meninges of mice. Bars, 500 μm. **(C)** Representative images are shown for GFP and PECAM immunostaining of meninges of *Prox1*^*GFP*^ mice. Bars, 500 μm. **(D)** Representative images are shown for LYVE-1 and PDPN immunostaining of meninges of mice. Bars, 1,000 μm. **(E,F)** Representative images are shown for GFP and LYVE-1 immunostaining of meninges of *Flt4*^*YFP*^ **(E)** and *Prox1*^*GFP*^ **(F)** mice. Bars, 500 μm. **(G)** Representative images are shown for LYVE-1 and F4/80 immunostaining of meninges of mice. Bars, 500 μm. **(H)** Representative native fluorescent stereo microscopic images are shown for native GFP signal in isolated meninges of *Prox1*^*GFP*^ and littermate control mice. Bars, 500 μm.

Supplementary Figure 2 Characterization of lymph nodes in *Plc*γ*2*^−/−^ and littermate control mice. **(A)** Representative stereo microscopic images of mesenterial lymph nodes, inguinal lymph nodes, superficial cervical lymph nodes and deep cervical lymph nodes of young adult *Plc*γ*2*^+/+^*, Plc*γ*2*^+/−^ and *Plc*γ*2*^−/−^ mice with mild or severe gut phenotype (*n* = 11 mice for *Plc*γ*2*^+/+^, *n* = 3 mice for *Plc*γ*2*^+/−^, *n* = 4 mice for *Plc*γ*2*^−/−^ mice with mild gut phenotype, *n* = 7 mice for *Plc*γ*2*^−/−^ mice with severe phenotype). Purple arrowheads point to lymph nodes. Bars, 1,000 μm. **(B)** Representative histological images of lymph nodes are shown by Haematoxylin-Eosin staining (*n* = 3 mice for *Plc*γ*2*^+/+^, *n* = 2 mice for *Plc*γ*2*^+/−^, *n* = 1 for *Plc*γ*2*^−/−^ mice with mild gut phenotype, *n* = 1 for *Plc*γ*2*^−/−^ mice with severe gut phenotype). Bars, 50 μm. **(C)** Representative images are shown for presence of erythrocytes in lymph nodes detected by fluorescent TER-119 immunostaining of lymph node samples isolated from young adult *Plc*γ*2*^+/+^*, Plc*γ*2*^+/−^ and *Plc*γ*2*^−/−^ mice with mild or severe gut phenotype (*n* = 3 mice for *Plc*γ*2*^+/+^, *n* = 2 mice for *Plc*γ*2*^+/−^, *n* = 1 for *Plc*γ*2*^−/−^ mice with mild gut phenotype, *n* = 1 for *Plc*γ*2*^−/−^ mice with severe gut phenotype). Bars, 50 μm.

## Abbreviations

CLEC2: C-type lectin domain family 1 member B
CNS: Central nervous system
E: Embryonic day
LEC: Lymphatic endothelial cell
LYVE-1: Lymphatic vessel endothelial hyaluronan receptor 1
P: Postnatal day
PBS: Phosphate buffered saline
PDPN: Podoplanin
PECAM: Platelet endothelial cell adhesion molecule
PFA: Paraformaldehyde
PLCγ2: Phospholipase C γ 2
PROX-1: Prospero homeobox 1
RhD: Rhodamine dextran
VEGFR3: Vascular endothelial growth factor receptor 3
VEGFC: Vascular endothelial growth factor C
WT: Wild type.
