# Supplementary figures and images for: Lymph Flow Induces the Postnatal Formation of Mature and Functional Meningeal Lymphatic Vessels
Source: Front Immunol. 2020 Jan 14;10:3043. doi: 10.3389/fimmu.2019.03043 (PMC6970982; doi:10.3389/fimmu.2019.03043)

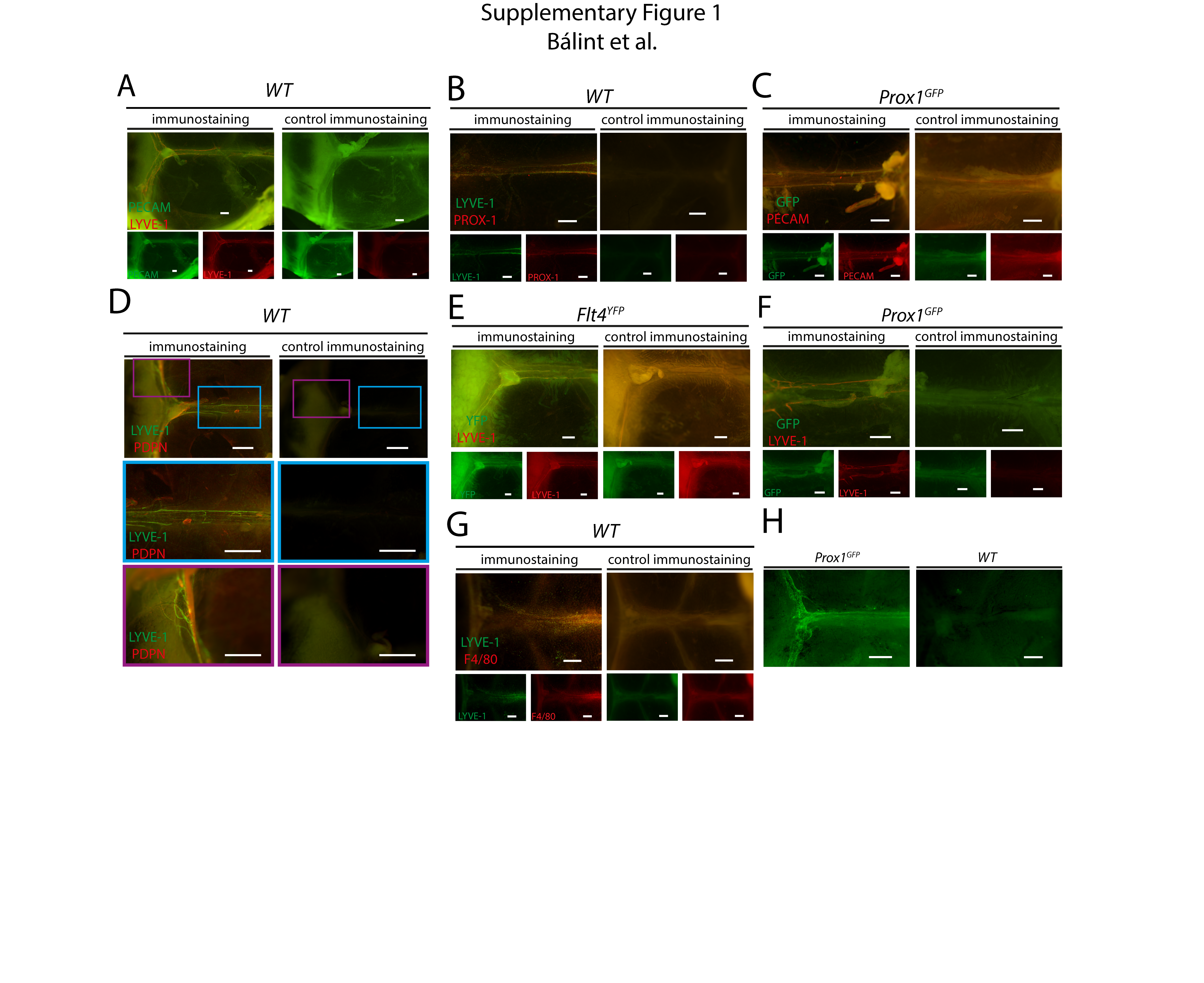

Supplement: Supplementary Figure 1 — Representative images of control immunostainings of meninges. Whole-mount immunostaining and control immunostaining of samples for LYVE-1, PDPN, PROX-1, PECAM, GFP and F4/80 in the dura mater of wild type, Prox1GFP and Flt4YFP mice. (A,B) Representative images are shown for PECAM and LYVE-1 immunostaining (A) and LYVE-1 and PROX-1 immunostaining (B) of meninges of mice. Bars, 500 μm. (C) Representative images are shown for GFP and PECAM immunostaining of meninges of Prox1GFP mice. Bars, 500 μm. (D) Representative images are shown for LYVE-1 and PDPN immunostaining of meninges of mice. Bars, 1,000 μm. (E,F) Representative images are shown for GFP and LYVE-1 immunostaining of meninges of Flt4YFP (E) and Prox1GFP (F) mice. Bars, 500 μm. (G) Representative images are shown for LYVE-1 and F4/80 immunostaining of meninges of mice. Bars, 500 μm. (H) Representative native fluorescent stereo microscopic images are shown for native GFP signal in isolated meninges of Prox1GFP and littermate control mice. Bars, 500 μm. [file Image_1.TIF]

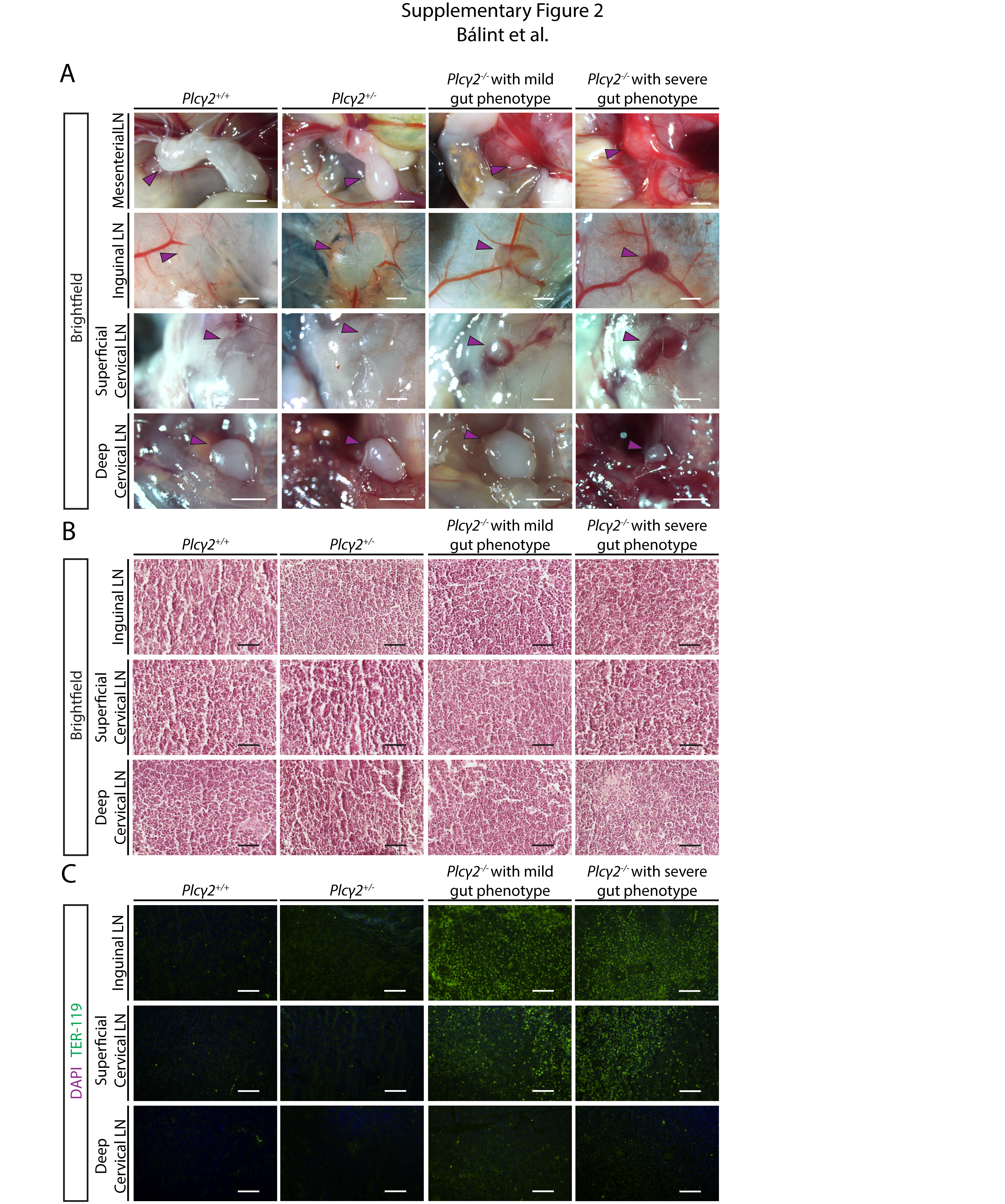

Supplement: Supplementary Figure 2 — Characterization of lymph nodes in Plcγ2−/− and littermate control mice. (A) Representative stereo microscopic images of mesenterial lymph nodes, inguinal lymph nodes, superficial cervical lymph nodes and deep cervical lymph nodes of young adult Plcγ2+/+, Plcγ2+/− and Plcγ2−/− mice with mild or severe gut phenotype (n = 11 mice for Plcγ2+/+, n = 3 mice for Plcγ2+/−, n = 4 mice for Plcγ2−/− mice with mild gut phenotype, n = 7 mice for Plcγ2−/− mice with severe phenotype). Purple arrowheads point to lymph nodes. Bars, 1,000 μm. (B) Representative histological images of lymph nodes are shown by Haematoxylin-Eosin staining (n = 3 mice for Plcγ2+/+, n = 2 mice for Plcγ2+/−, n = 1 for Plcγ2−/− mice with mild gut phenotype, n = 1 for Plcγ2−/− mice with severe gut phenotype). Bars, 50 μm. (C) Representative images are shown for presence of erythrocytes in lymph nodes detected by fluorescent TER-119 immunostaining of lymph node samples isolated from young adult Plcγ2+/+, Plcγ2+/− and Plcγ2−/− mice with mild or severe gut phenotype (n = 3 mice for Plcγ2+/+, n = 2 mice for Plcγ2+/−, n = 1 for Plcγ2−/− mice with mild gut phenotype, n = 1 for Plcγ2−/− mice with severe gut phenotype). Bars, 50 μm. [file Image_2.TIF]
